# Design, sythesis and evaluation of a series of 3- or 4-alkoxy substituted phenoxy derivatives as PPARs agonists

**DOI:** 10.18632/oncotarget.15198

**Published:** 2017-02-08

**Authors:** Jun Zhang, Xue-Jiao Wang, Xin Liu, Yi Huan, Miao-Miao Yang, Zhu-Fang Shen, Wen-Qing Jia, Zhi Jing, Shu-Qing Wang, Wei-Ren Xu, Xian-Chao Cheng, Run-Ling Wang

**Affiliations:** ^1^ Tianjin Key Laboratory on Technologies Enabling Development of Clinical Therapeutics and Diagnostics (Theranostics), School of Pharmacy, Tianjin Medical University, Tianjin 300070, China; ^2^ Institute of Materia Medica, Chinese Academy of Medical Sciences and Peking Union Medical College, Beijing 100050, China; ^3^ Tianjin Key Laboratory of Molecular Design and Drug Discovery, Tianjin Institute of Pharmaceutical Research, Tianjin 300193, China

**Keywords:** PPARs, Type 2 diabetes mellitus, bioisosterism, docking analysis, molecular dynamic simulations

## Abstract

Peroxisome proliferators-activated receptors (PPARα, γ and δ) are potentially effective targets for Type 2 diabetes mellitus therapy. The severe effects of known glitazones and the successfully approved agents (saroglitazar and lobeglitazone) motivated us to study novelly potent PPARs drugs with improved safety profile. In this work, we received 15 carboxylic acids based on the combination principle to integrate the polar head of bezafibrate with the hydrophobic tail of pioglitazone. Another 12 tetrazoles based on the bioisosterism principle were obtained accordingly. Furthermore, *in vitro* PPARs transactivation assays on these 3- or 4-alkoxy substituted phenoxy derivatives afforded six compounds. Interactions and binding stability from the docking analysis and 20 ns molecular dynamic simulations confirmed the representative compounds to be suitable and plausible for PPARs pockets. The above-mentioned results demonstrated that the compounds may be used as reference for further optimization for enhanced PPARs activities and wide safety range.

## INTRODUCTION

**Figure 1:**
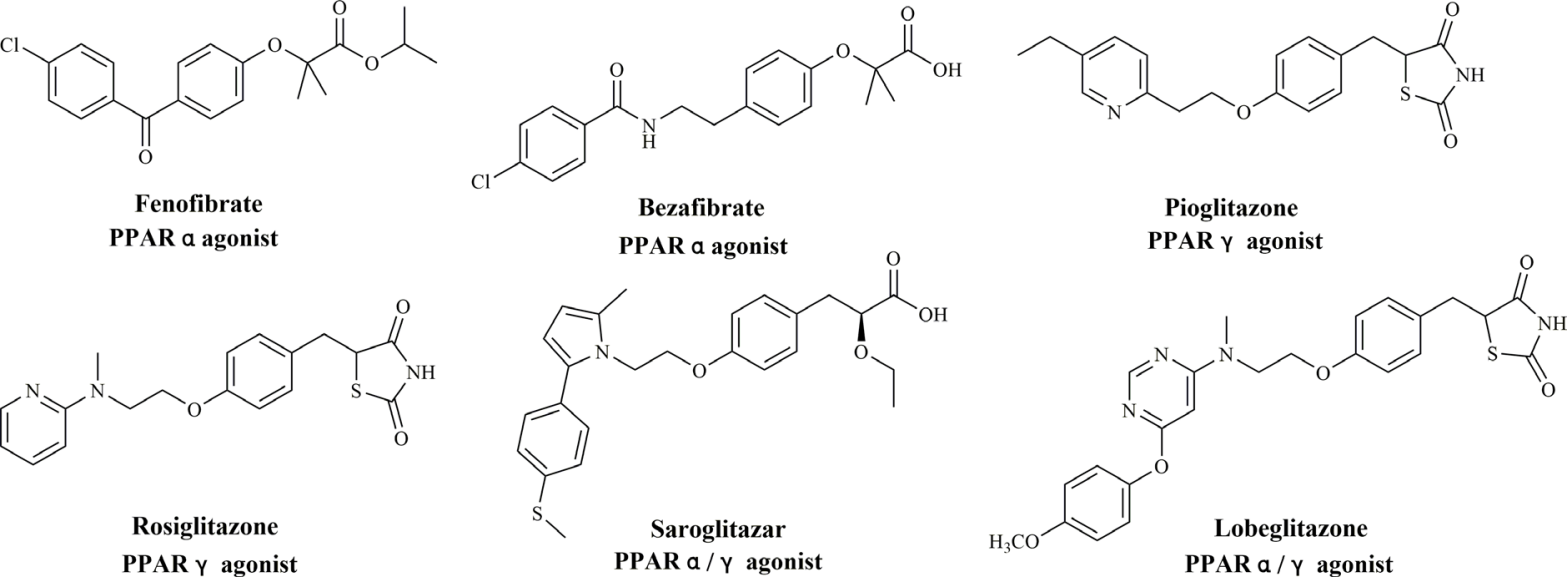
Structures of PPARs ligands

Peroxisome proliferators-activated receptors (PPARα, γ and δ) are classified as ligand-inducible nuclear receptors [1, 2]. They were attractive targets for diabetes, dyslipidemia, obesity, inflammation and atherosclerosis [3]. These identified subtypes displayed high similarity in sequence and significant specificity in physiological functions and tissue distribution. PPARα is highly expressed in liver, kidney, heart, skeletal muscle and adipose tissue [4]. When activated by ligands, fatty acid oxidation and lipoprotein metabolism would be improved. Drugs targeting PPARα in market are fibrates such as fenofibrate and bezafibrate (Figure 1), which are mainly served as hypolipidemic drugs to treat hypertriglyceridemia [5]. PPARγ which highly ameliorates insulin sensitivity is widely expressed in adipose tissues [6–8]. Moreover, the fatty acid precipitation and decomposition would be accelerated through PPARγ activation. PPARγ ligands glitazones (rosiglitazone and pioglitazone, Figure 1) are deemed as insulin sensitizer to improve the symptoms of patients with Type 2 diabetes mellitus (T2DM) [9]. PPARδ, however, is expressed in ubiquity in almost all tissues to fasten the metabolism of lipids and sugars [10, 11]. Nevertheless, there are no marketed drugs targeting PPARδ available. GW501516 which exhibited potent and selective PPARδ agonism has previously entered clinical trials [12].

However, despite of the potent activity, glitazones were discontinued in clinic due to the serious side effects such as weight gain, edema, hepatotoxicity and cardiovascular toxicity [9, 13, 14]. The primary reason may attribute to the single full or partial PPARs activation. Evidences from previous studies indicated that the roles of multi-targets would be beneficial to the pharmacological activities. Saroglitazar [15] and lobeglitazone [16] (Figure 1) with dual PPARα/γ agonism have been approved by Drug Controller General of India and Korean Food and Drug Administration, respectively. Both agents exhibited excellent profile in glucose and lipids level and wide safety range in the treatment of T2DM.

**Figure 2:**
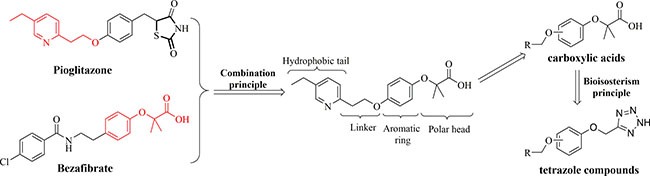
The design process of 3- or 4-alkoxy substituted phenoxy derivatives

Motivated by the hot concept of multi-targets, we aimed to design novel PPARs agents based on the structures of reported PPARs drugs. In this study, we utilized the combination principle to integrate the polar head of bezafibrate with the hydrophobic tail of pioglitazone (Figure 2). Various alkoxy groups were substituted in the *meta*- or *para*-position to afford 15 carboxylic acids initially. Another 12 compounds replaced with a tetrazole ring were produced according to the bioisosterism principle [17–19]. Therefore, 27 combinatorial molecules with the main structural skeleton of 3- or 4-alkoxy substituted phenoxyl were available as potential PPARs agents. The design process was listed as Figure 2. In further bioactivity evaluation on PPARs activation, six compounds were originally found with PPARs activation.

Herein, we carried out our work with 27 compounds for design, synthesis and biological evaluation details. Recent docking analysis and molecular dynamics (MD) simulations of 20 ns validated the binding modes and dynamics stability of best docked complexes with PPARs.

## RESULTS AND DISCUSSION

### Synthesis

**Scheme 1:**
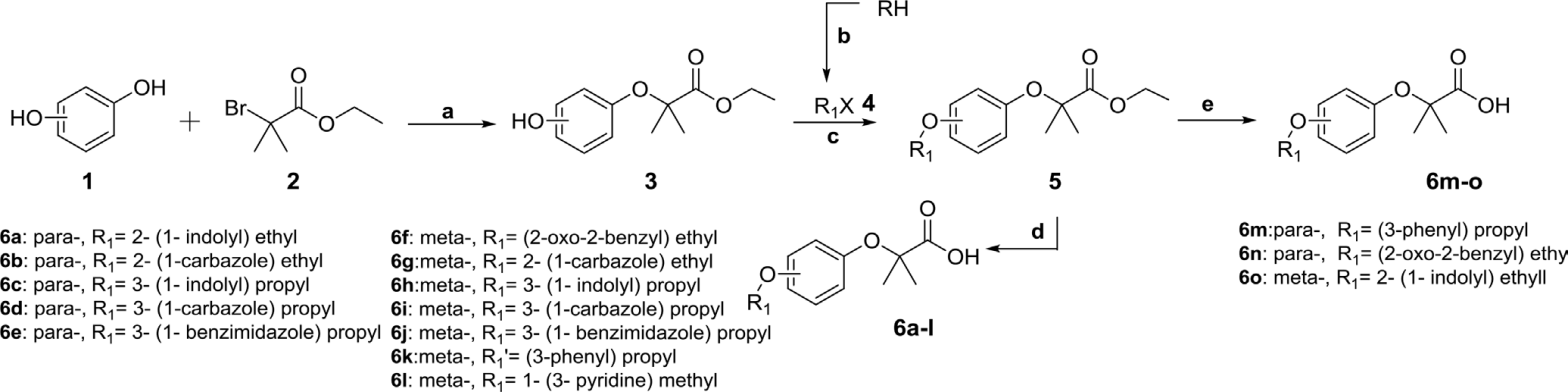
Reagents and conditions (**A**) acetone, K_2_CO_3_, NaI, at 65°C; (**B**) KOH, K_2_CO_3_, TBAB, 1, 2-dihalogen ethane or 1, 3-dihalogen propane, water, at 45°C; (**C**) acetone, K_2_CO_3_, NaI, at 65°C; (**D**) THF-CH_3_OH-water (3:1:1), LiOH, hydrochloric acid; (**E**) CH_3_OH, NaOH, at 50°C, hydrochloric acid.

**Scheme 2:**
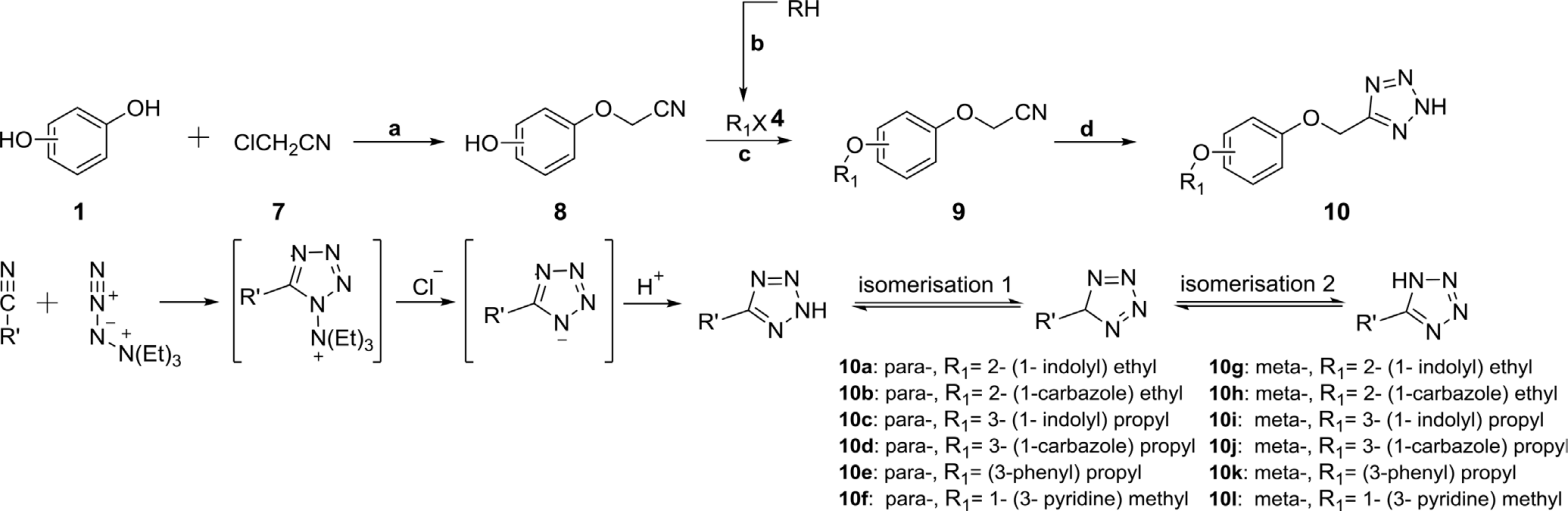
Reagents and conditions (**A**) acetone, K_2_CO_3_, NaI, at 65°C; (**B**) KOH, K_2_CO_3_, TBAB, 1,2-dihalogen ethane or 1,3-dihalogen propane, water, at 45°C; (**C**) acetone, K_2_CO_3_, NaI, at 65°C; (**D**) toluene, (Et)_3_N·HCl, NaN_3_, at 120°C, hydrochloric acid.

The general synthetic routes of carboxylic acids and tetrazole compounds were depicted in Schemes 1 and 2, respectively.

With hydroquinone and resorcinol (1) respectively as raw materials, one important monosubstituted intermediate 3 was obtained through the nucleophilic substitution reaction with ethyl 2-bromo-2-methylpropanoate (Scheme 1), while the other unwanted disubstituted by-product was removed by flash column chromatography method. The esters 5 were synthesized via Williamson ether synthesis reaction with various halogenated organic compounds 4. Under basic conditions, the final products of *para*- and *meta*-series (6) were received via the hydrolysis reaction to 5 [20]. Equally as shown in Scheme 2, intermediates 8 and 9 were successfully synthesized via the nucleophilic substitution reaction. Using toluene as the solvent, compounds 9 were reacted with triethylamine hydrochloride and sodium azide. Through dipolar cycloaddition, we afforded target tetrazoles of *para*- and *meta*-series (10) [21]. Additionally, the tautomerism occurred to the tetrazole molecules as indicated in Scheme 2.

### *In vitro* PPARα/γ/δ transactivation assays

As indicated in introduction section, we successfully synthesized 27 combinatorial molecules with the main structural skeleton of 3- or 4-alkoxy substituted phenoxyl. Three different PPARs (α, γ and δ) *in vitro* transaction assays were introduced to these molecules under the constant concentration (10^−5^ M). The relative activities were respectively compared to the positive controls. Supplementary Table 1 listed the structures and *in vitro* preliminary evaluation results of 3- or 4-alkoxy substituted phenoxy derivatives towards PPARs activation. After preliminary biological evaluation to these 27 compounds, six were examined and screened with potential PPARs agonistic activities (Table 1). Molecule 6h exhibited weaker PPARα activation (48.5%) compared to the positive control, 6g and 10h weakly activated PPARγ by 24.4% and 35.8%, respectively while compounds 6e (63.8%), 6l (58.6%) and 10l (89.7%) demonstrated medium intensity or high potency in PPARδ activation. In further evaluation under various concentrations, these six molecules with potential PPARs affinities were investigated through median effective concentration (EC_50_) and concentration at maximum efficiency percentage (C_max_). GW7647, rosiglitazone and GW501516 were selected as positive controls.

**Table 1:** The PPARs activation values of compounds from preliminary screening

	PPARα		PPARγ			PPARδ			
	6h	GW7647	6g	10h	rosiglitazone	6e	6l	10l	GW501516
EC_50_^a^	5.30 ×10^−6^	9.44 ×10^−9^	1.18 ×10^−5^	6.97 ×10^−6^	3.954 ×10^−6^	1.11 ×10^−8^	1.90 ×10^−9^	2.73 ×10^−8^	4.293×10^−9^
C_max_^b^	1 ×10^−5^	1 ×10^−7^	5 ×10^−5^	3 ×10^−5^	1 ×10^−4^	1 ×10^−7^	1 ×10^−7^	1 ×10^−7^	1×10^−5^

a: EC_50_ (M), median (50%) effective concentration; b: C_max_ (M), concentration at maximum efficiency percentage (Max%).

**Figure 3:**
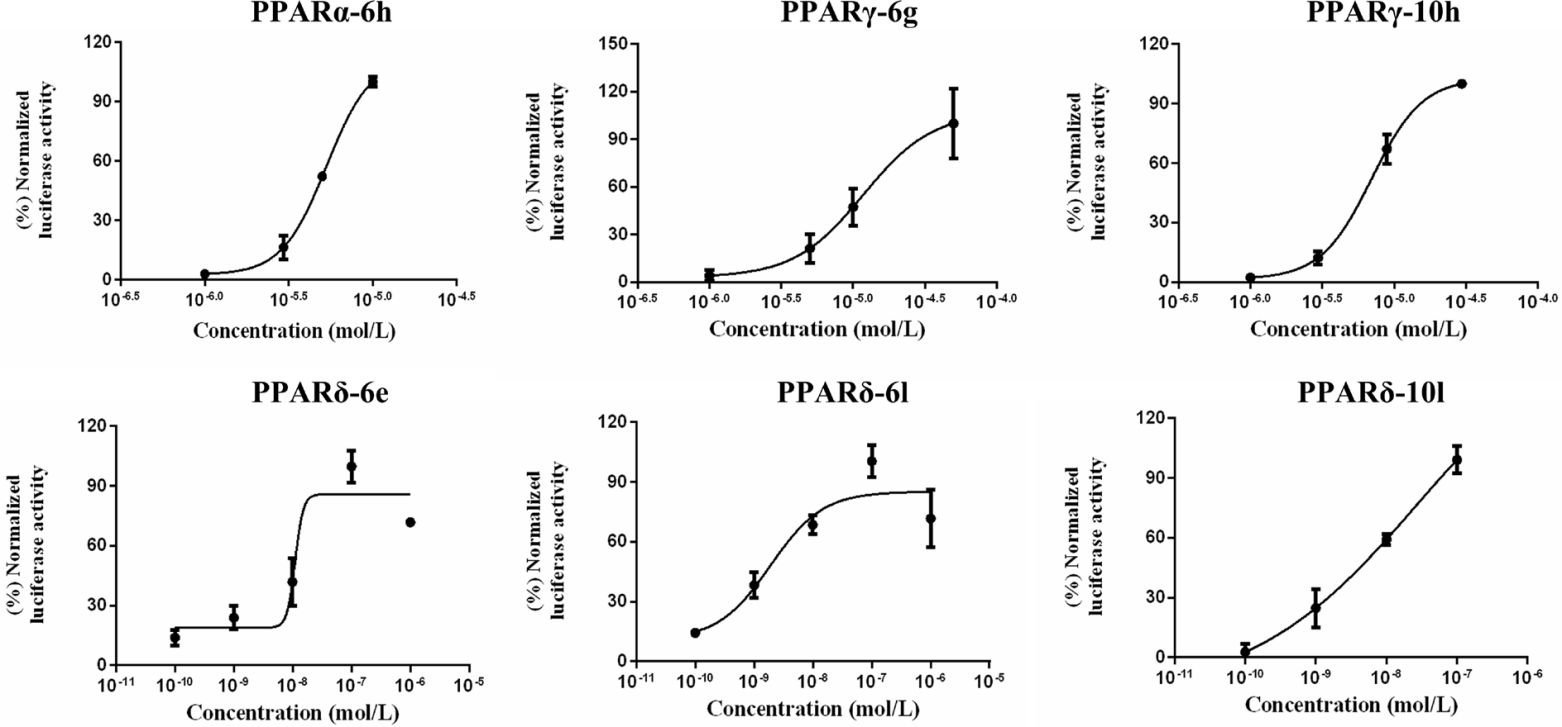
The concentration- relative activity curves of screened molecules activating PPARs

According to the relative activities of six screened compounds under various concentrations, EC_50_s and C_max_s values of molecules in activating PPARs were deduced in the concentration-relative activity curves (Figure 3). Clearly in Table 1, compound 6h activated PPARα with an EC_50_ value of 5.30 × 10^−6^ M and attained the maximum efficiency percentage at the concentration of 1 × 10^−5^ M. When compared to GW7647, the effect-acting concentration of 6h was relatively higher and a significant discrepancy in efficiency existed in both compounds. Identically in activating PPARγ, target compounds 6g and 10h took the EC_50_s of 1.18×10^−5^ M and 6.97 × 10^−6^ M, respectively. However, their concentrations for maximum efficiency percentage (5 × 10^−5^ M and 3 × 10^−5^ M, respectively) were some lower than rosiglitazone. As for PPARδ activation, compounds 6e, 6l and 10l exhibited lower C_max_ values and agonistic activities with an extent close to the control, illustrating their moderate or strong agonism towards PPARδ.

### Molecular docking

**Figure 4:**
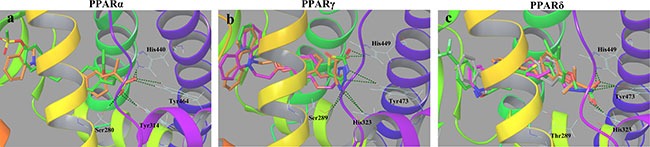
The putative binding behaviours from docking analysis (A) AZ242 (green) and 6h (orange) in PPARα-LBD (PDB ID:1I7G). (B) rosiglitazone (green), 6g (orange) and 10h (purple) in PPARγ-LBD (PDB ID:2PRG). (C) D32 (green), 6e (gray), 6l (orange) and 10l (purple) in PPARδ-LBD (PDB ID:3GZ9).

To confirm the PPARs activities of known 6h, 6g, 10h, 6e, 6l and 10l, we conducted the docking analysis to investigate the interactions with the corresponding receptor. The interaction behaviors between the reference drugs and PPARs active sites were shown in Supplementary Figures 1 and 2. Hydrogen bonds and hydrophobic contacts were essential to the docking stability and binding affinity in protein-ligand complexes. As indicated in Figure 4, compounds 6h (PPARα), 6g and 10h (PPARγ), 6e, 6l and 10l (PPARδ) fitted well into PPARs active cavities where the original ligands bound, demonstrating their potentially strong binding affinity. Additionally, these compounds aligned very compactly and properly with the correspondingly original ligands, especially in polar head and hydrophobic regions. The binding modes were represented to be plausible and similar to those of co-crystal ligands.

The polar heads (carboxylic acid and tetrazole ring) of synthetic molecules were oriented towards AF-2 domain in H12 and interacted with key amino acids through the H-bonds networks as AZ242 in PPARα-LBD, rosiglitazone in PPARγ-LBD and D32 in PPARδ-LBD. The hydrophobic groups of 6h, 6g, 10h, 6e, 6l and 10l were similarly located in the hydrophobic area of PPARs-LBD. According to the aforementioned results, the excellent interaction modes may exactly interpret the results from *in vitro* PPARs avtivities.

### Molecular dynamics simulation

**Figure 5:**
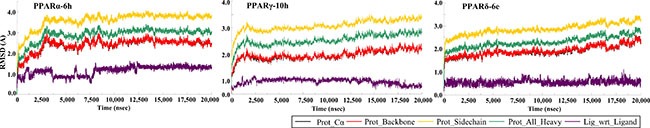
The RMSD trajectories of PPARα-6h, PPARγ-10h and PPARδ-6e complexes throughout 20 ns simulations ‘Lig_wrt_Ligand’ meant the ligand aligned on itself.

**Figure 6:**
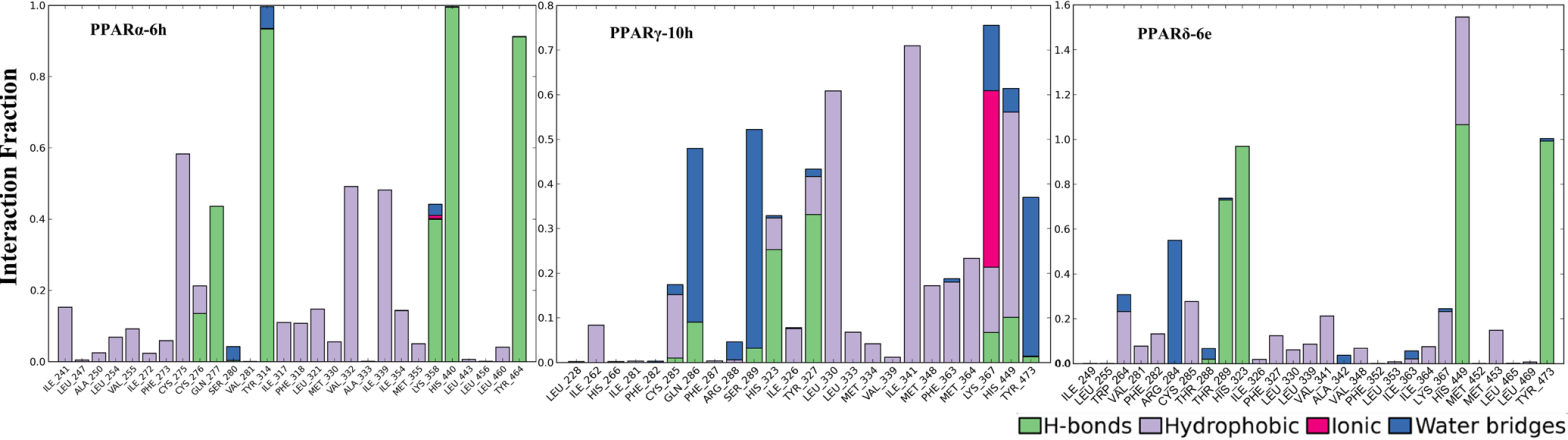
The bar graphs of protein-ligand (PL) contacts (PPARα-6h, PPARγ-10h and PPARδ-6e) Colored bar graphs of green, lavender, red and blue represented H-bonds, hydrophobic, ionic contacts and water bridges, respectively.

20 ns simulaions performed with Desmond v4.3 (D.E. Shaw Research, New York, NY, 2015) program were utilized to evaluate the binding stability in dynamics state. The RMSD trajectories of PPARα-6h, PPARγ-10h and PPARδ-6e complexes throughout 20 ns simulations (Figure 5) illustrated the conformations to be excellent. Relatively, the complexes tended to be in equilibrium, indicating stable binding conformations in dynamics environment. The ligand itself (6h, 10h and 6e) retained roughly unchanged over the entire simulations time. Additionally in Figure 6, the interactions of molecules with PPARs pocket listed detailed fractions of residues. Obviously, the binding stability was available through the H-bonds, hydrophobic, ionic contacts and water bridges between molecules and proteins. In PPARα-LBD, 6h attained binding stability through the H-bonds interactions of the polar head with key amino acids (Tyr314, His440 and Tyr464) and other hydrophobic contacts. Equally with PPARγ-10h, H-bonds interactions and hydrophobic contacts with larger fractions (Ser289, Tyr327, His449 and Tyr473) contributed to the stability. As for PPARδ-6e, the higher interaction fractions of Thr289, His323, His449 and Tyr473 exactly explained the relatively higher PPARδ agonistic activity as indicated in PPARs *in vitro* activation assays.

## MATERIALS AND METHODS

### Chemistry

All the reagents used in the experiments were analytically pure and purchased from assigned commercial suppliers. The X-6 micro melting point apparatus was ulitized to measure the melting points (m.p.). Through the thin-layer chromatography (TLC) method, the silica gel plates viewed under the box type automatic UV analyzer (ZF-2C) (254 nm) was to determine the process of the reaction. The ^1^H-NMR and ^13^C-NMR spectra of new products that dissolved in CDCl_3_ or DMSO-*d*_6_ solvent recorded the chemical shifts of H and C by a Bruker spectrometer with TMS as the standard material. The molecular weight of one compound was confirmed with the mass spectrometer.

### *Para*-/*meta*- monosubstituted intermediates (3)

Hydroquinone / resorcinol 1 (2.2 g, 20 mmol) was dissolved in the solution of acetone (50 ml), followed by K_2_CO_3_ (2.76 g, 20 mmol) and NaI (catalytic amounts). Then a mixed solution of ethyl 2-bromo-2-methylpropanoate 2 (1.95 g, 10 mmol) and acetone (50 ml) was dripped slowly to the mixture above. The reaction was heated to reflux at 65°C for 48 h under stirring condition. The process of the reaction was detected through TLC method. After the reaction, crude products were obtained through the course of filtration and vacuum distillation to remove the solvent. The final products (3) were purified by flash column chromatography with petroleum ether/EtOAc as the mobile phase.

3a (Ethyl 2-(4-hydroxyphenoxy)-2-methyl propanoate): white solid (33%), R_f_ = 0.70 (developing agent: dichloromethane / acetone = 100:1); ^1^H-NMR (CDCl_3_, 400MHz): δ 6.78 (2H, q, *J* = 2.98Hz, Ar-H); 6.69 (2H, q, *J* = 2.98Hz, Ar-H); 5.30 (1H, s, OH); 4.24 (2H, q, *J* = 7.13Hz, CH_2_); 1.53 (6H, s, CH_3_); 1.28 (3H, t, *J* = 7.12 Hz, CH_3_). MS *m/z*: 470.93 (2M+Na).

3b (Ethyl 2-(3-hydroxyphenoxy)-2-methyl propanoate): colorless oil (11.35%), R_f_ = 0.48 (developing agent: dichloromethane / acetone = 20:1); ^1^H-NMR (CDCl_3_, 400MHz): δ 7.050-7.029 (1H, d, *J* = 8.4Hz, Ar-H); 6.496-6.468 (1H, m, Ar-H); 6.396-6.375 (2H, m, Ar-H); 6.300-6.100 (1H, s, OH); 4.257-4.204 (2H, m, CH_2_); 1.585 (6H, s, CH_3_); 1.256-1.220 (3H, m, CH_3_). ^13^C-NMR (CDCl_3_, δ): 174.93 (C=O), 156.73 (C aro), 156.48 (C aro), 129.74 (C aro), 111.16 (C aro), 109.52 (C aro), 106.71 (C aro), 79.23 (C(CH_3_)_2_), 61.76 (CH_2_), 25.34 (2×CH_3_), 13.98 (CH_3_). MS *m/z*: 223.13 (M-1), 247.13 (M+Na), 447.07 (2M-1), 470.93 (2M+Na).

### Halogenated organic compounds (4)

N-Heterocycle (15 mmol), KOH (8.4 g, 150 mmol), K_2_CO_3_ (20 g, 150 mmol), TBAB (0.48 g, 1.5 mmol) were dissolved in 100 ml 1, 2-dihalogen ethane or 1, 3-dihalogen propane. The mixture was heated to reflux at 45°C for 24 h. The process of the reaction was detected through TLC method. After the reaction, anhydrous Na_2_SO_4_ was used to dehydrate the organic layer from a separatory funnel. The crude products were obtained through the course of filtration and vacuum distillation to remove the solvent. The final products (4) were purified by flash column chromatography with petroleum ether/EtOAc as the mobile phase.

4a (1-(2-Chloroethyl)-1H-indole): colorless oil (24%), R_f_ = 0.55 (developing agent: petroleum ether/EtOAc = 20:1); ^1^H-NMR (CDCl_3_, 400MHz): δ 8.136 (1H, m, Ar-H); 7.688-7.583 (3H, m, Ar-H); 7.362 (1H, s, Ar-H); 6.964 (1H, s, Ar-H); 4.482-4.450 (2H, t, *J* = 6.4Hz, CH_2_); 3.915-3.884 (2H, t, *J* = 6.4Hz, CH_2_). ^13^C-NMR (CDCl_3_, δ): 136.12 (C aro), 129.14 (C aro), 128.59 (C aro), 122.20 (C aro), 121.56 (C aro), 120.17 (C aro), 109.44 (C aro), 102.16 (C aro), 48.02 (CH_2_), 43.04 (CH_2_). MS *m/z*: 361.20 (2M+1).

4b (9-(2-Chloroethyl)-9H-carbazole): white flocculus (30%), m.p. 127.6-128.8°C, R_f_ = 0.53 (developing agent: petroleum ether / EtOAc = 10:1); ^1^H-NMR (CDCl_3_, 400MHz): δ 8.106 (2H, m, Ar-H); 7.501-7.425 (4H, m, Ar-H); 7.282-7.243 (2H, m, Ar-H); 4.646 (2H, t, *J* = 7.2Hz, CH_2_); 3.860 (2H, t, *J* = 7.2Hz, CH_2_). ^13^C-NMR (CDCl_3_, δ): 140.13 (2×C aro), 125.95 (2×C aro), 123.13 (2×C aro), 120.53 (2×C aro), 119.54 (2×C aro), 108.46 (2×C aro), 44.73 (CH_2_), 41.00 (CH_2_).

4c (1-(3-Chloropropyl)-1H-indole): colorless oil (44.52%), R_f_ = 0.70 (developing agent: petroleum ether / EtOAc = 10:1); ^1^H-NMR (CDCl_3_, 400MHz): δ 7.673-7.632 (1H, m, Ar-H); 7.363-7.393 (1H, m, =CH); 7.256-7.199 (1H, m, Ar-H); 7.136-7.093 (2H, m, Ar-H); 6.511(1H, s, =CH); 4.363-4.331 (2H, t, *J* = 6.4Hz, CH_2_); 3.470-3.440 (2H, t, *J* = 6.0Hz, CH_2_); 2.306-2.244 (2H, m, CH_2_). ^13^C-NMR (CDCl_3_, δ): 135.85 (C aro), 128.72 (C aro), 128.00 (=CH), 121.65 (C aro), 121.08 (C aro), 119.49 (C aro), 109.22 (C aro), 101.52 (=CH), 42.87 (CH_2_), 41.85 (CH_2_) , 32.64 (CH_2_).

4d (9-(3-Chloropropyl)-9H-carbazole): colorless oil (40%), R_f_ = 0.70 (developing agent: dichloromethane/acetone = 10:1); ^1^H-NMR (CDCl_3_, 400MHz): δ 8.104-8.085 (2H, d, *J* = 7.6Hz, Ar-H); 7.466 (4H, s, Ar-H); 7.258-7.228 (2H, m, Ar-H); 4.509-4.477 (2H, t, *J* = 6.4Hz, CH_2_); 3.519-3.490 (2H, t, *J* = 6.0Hz,, CH_2_); 2.362-2.300 (2H, m, CH_2_). ^13^C-NMR (CDCl_3_, δ): 140.40 (C aro), 126.13 (2×C aro), 123.00 (C aro), 120.42 (2×C aro), 119.15 (3×C aro), 108.57 (3×C aro), 55.07 (CH_2_), 42.31 (CH_2_), 31.80 (CH_2_).

4e (1-(3-Bromopropyl)-1H-indole): colorless oil (68.27%), R_f_ = 0.55 (developing agent: dichloromethane /acetone = 10:1); ^1^H-NMR (CDCl_3_, 400MHz): δ 7.642-7.623 (1H, d, *J* = 7.6Hz, Ar-H); 7.381-7.361 (1H, d, *J* = 8.0Hz, Ar-H); 7.234-7.178 (1H, m, =CH); 7.142-7.084 (2H, m, Ar-H); 6.505 (1H, s, =CH); 4.340-4.265 (2H, m, CH_2_); 3.308-3.277 (2H, t, *J* = 6.4Hz, CH_2_) ; 2.369-2.160 (2H, m, CH_2_). ^13^C-NMR (CDCl_3_, δ): 136.84 (C aro), 128.74 (C aro), 128.05 (C aro), 121.67 (C aro), 120.95 (C aro), 119.53 (C aro) , 109.58 (C aro), 101.55 (C aro), 43.98 (CH_2_) , 32.74 (CH_2_), 30.96 (CH_2_).

4f (9-(3-Bromopropyl)-9H-carbazole): colorless oil (73.7%), R_f_ = 0.50 (developing agent: dichloromethane/acetone = 10:1); ^1^H-NMR (CDCl_3_, 400MHz): δ 8.110-8.090 (2H, d, *J* = 8.0Hz, Ar-H); 7.497-7.483 (4H, d, *J* = 5.6Hz, Ar-H); 7.298-7.244 (2H, m, Ar-H); 4.512-4.480 (2H, t, *J* = 6.4Hz, CH_2_); 3.399-3.368 (2H, t, *J* = 6.0Hz, CH_2_); 2.467-2.405 (2H, m, CH_2_). ^13^C-NMR (CDCl_3_, δ): 140.38 (C aro), 132.37 (2×C aro), 126.16 (C aro), 123.01 (2×C aro), 120.47 (2×C aro), 119.19 (2×C aro), 108.82 (2×C aro), 45.30 (CH_2_), 32.78 (CH_2_), 30.98 (CH_2_).

4g (1-(3-Chloropropyl)-1H-benzo[d]imidazole): colorless oil (21.54%), R_f_ = 0.48 (developing agent: dichloromethane / acetone = 10:1); ^1^H-NMR (CDCl_3_, 400MHz): δ 8.020 (1H, s, =CH); 7.845-7.823 (1H, m, Ar-H); 7.461-7.428 (1H, m, Ar-H); 7.355-7.260 (2H, m, Ar-H); 4.450-4.417 (2H, t, *J* = 6.4Hz, CH_2_); 3.506-3.476 (2H, t, *J* = 6.0Hz, CH_2_); 2.369-2.306 (2H, m, CH_2_). ^13^C-NMR (CDCl_3_, δ): 143.48 (C aro), 143.02 (C aro), 133.51 (C aro), 123.27 (C aro), 122.50 (C aro), 120.46 (C aro), 109.53 (C aro), 60.38 (CH_2_), 41.22 (CH_2_), 32.07 (CH_2_).

### Ester compounds 5

Monosubstituted intermediates 3 (2.24 g, 10 mmol), halogenated organic compounds 4 (10 mmol), K_2_CO_3_ (3.32 g, 24 mmol) and NaI (catalytic amounts) were added to 50 ml acetone. The solution was heated to reflux at 65°C for 24 h under stirring condition. The process of the reaction was detected through TLC method. After the reaction, the crude esters were obtained through the course of filtration and vacuum distillation to remove the solvent. The final products 5 were purified by flash column chromatography with petroleum ether/EtOAc as the mobile phase.

5a (Ethyl 2-methyl-2-(4-(2-oxo-2-phenylethoxy)phenoxy)propanoate): colorless oil (39.77%), R_f_ = 0.45 (developing agent: petroleum ether / EtOAc = 10:1); ^1^H-NMR (CDCl_3_, 400MHz): δ 7.974 (2H, m, Ar-H); 7.596 (1H, d, *J* = 7.2Hz, Ar-H); 7.478 (2H, d, *J* = 7.6Hz, Ar-H); 6.829-6.772 (4H, m, Ar-H); 5.210 (2H, s, CH_2_); 4.248-4.194 (2H, m, CH_2_); 1.538 (6H, s, CH_3_); 1.277-1.242 (3H, m, CH_3_). ^13^C-NMR (CDCl_3_, δ): 194.67 (C=O), 174.13 (C=O), 153.60 (C aro), 149.80 (C aro), 134.59 (C aro), 133.86 (C aro), 128.83 (2×C aro), 128.13 (2×C aro), 121.52 (2×C aro), 115.40 (2×C aro), 79.65 (C(CH_3_)_2_), 71.46 (CH_2_), 61.41 (CH_2_), 25.30 (2×CH_3_), 14.11 (CH_3_). MS *m/z*: 365.13 (M+Na).

5b (Ethyl 2-(4-(2-(1H-indol-1-yl)ethoxy)phenoxy)-2-methylpropanoate): colorless oil (22.7%), R_f_ = 0.40 (developing agent: petroleum ether / EtOAc = 10:1); ^1^H-NMR (CDCl_3_, 400MHz): δ 7.637-7.617 (1H, d, *J* = 8.0Hz, Ar-H); 7.399-7.377 (1H, d, *J* = 8.4Hz, = CH); 7.248-7.198 (2H, m, Ar-H); 7.127-7.107 (1H, m, Ar-H); 6.802-6.708 (3H, m, Ar-H); 7.399-7.377 (1H, m, Ar-H); 6.509-6.500 (1H, m, = CH); 4.497-4.469 (2H, t, *J* = 5.6Hz, CH_2_); 4.242-4.189 (4H, m, CH_2_); 1.579 (6H, s, CH_3_); 1.274-1.239 (3H, m, CH_3_). ^13^C-NMR (CDCl_3_, δ): 174.27 (C=O), 153.88 (C aro), 149.38 (C aro), 136.08 (C aro), 128.67 (C aro), 128.40 (C aro), 121.59 (2×C aro), 121.04 (C aro), 120.08 (C aro), 119.49 (C aro), 114.95 (2×C aro), 109.20 (C aro), 101.60 (C aro), 79.69 (C(CH_3_)_2_), 67.26 (CH_2_), 61.34 (CH_2_), 45.72 (CH_2_), 25.36 (2×CH_3_), 14.13 (CH_3_). MS *m/z*: 390.20 (M+Na).

5c (Ethyl 2-(4-(2-(9H-carbazol-9-yl)ethoxy)phenoxy)-2-methylpropanoate): colorless oil (6.2%), R_f_ = 0.47 (developing agent: petroleum ether/EtOAc = 10:1); ^1^H-NMR (CDCl_3_, 400MHz): δ 8.100 (1H, m, Ar-H); 7.507-7.450 (3H, dd, *J* = 8.4Hz, Ar-H); 7.260-7.225 (5H, m, Ar-H); 6.780-6.758 (1H, d, *J* = 8.8Hz, Ar-H); 6.687-6.666 (2H, d, *J* = 8.4Hz, Ar-H); 4.711-4.681 (2H, t, *J* = 6.0Hz, CH_2_); 4.310-4.116 (4H, m, CH_2_); 1.541 (6H, s, CH_3_); 1.278-1.233 (3H, m, CH_3_). ^13^C-NMR (CDCl_3_, δ): 174.25 (C=O), 153.89 (C aro), 140.54 (C aro), 125.72 (3×C aro), 123.00 (C aro), 121.54 (2×C aro), 120.34 (3×C aro), 119.16 (3×C aro),114.78 (2×C aro), 108.76 (2×C aro), 79.66 (C(CH_3_)_2_), 66.25 (CH_2_), 61.33 (CH_2_), 42.56 (CH_2_), 25.24 (2×CH_3_), 14.11 (CH_3_). MS *m/z*: 440.20 (M+Na).

5d (Ethyl 2-(4-(3-(1H-indol-1-yl)propoxy)phenoxy)-2-methylpropanoate): colorless oil (35.72%), R_f_ = 0.36 (developing agent: petroleum ether / EtOAc = 10:1); ^1^H-NMR (CDCl_3_, 400MHz): δ 7.628-7.609 (1H, d, *J* = 7.6Hz, Ar-H); 7.359-7.339 (1H, d, *J* = 8.0Hz, =CH); 7.184-7.146 (1H, t, *J* = 7.2Hz, Ar-H); 7.102-7.069 (2H, m, Ar-H); 6.839-6.808 (2H, m, Ar-H); 6.758-6.736 (2H, m, Ar-H); 6.472-6.465 (1H, m, =CH); 4.372-4.339 (2H, t, *J* = 6.4Hz, CH_2_); 4.265-4.212 (2H, m, CH_2_); 3.835-3.807 (2H, t, *J* = 5.6Hz, CH_2_); 2.283-2.060 (2H, m, CH_2_); 1.538 (6H, s, CH_3_); 1.296-1.261 (3H, t, *J* = 6.8Hz, CH_3_). ^13^C-NMR (CDCl_3_, δ): 174.28 (C=O), 154.31 (C aro), 149.21 (C aro), 135.95 (C aro), 128.84 (C aro), 128.07 (C aro), 127.69 (=CH), 121.63 (2×C aro), 120.97 (C aro), 119.41 (C aro), 114.91 (2×C aro), 109.30 (C aro), 101.49 (=CH), 79.71 (C(CH_3_)_2_), 64.74 (CH_2_), 61.49 (CH_2_), 42.97 (CH_2_), 29.94 (CH_2_), 25.31 (2×CH_3_), 14.11 (CH_3_). MS *m/z*: 382.27 (M+1), 404.27 (M+Na).

5e (Ethyl 2-(4-(3-(9H-carbazol-9-yl)propoxy)phenoxy)-2-methylpropanoate): colorless oil (15.84%), R_f_ = 0.39 (developing agent: petroleum ether / EtOAc = 5:1); ^1^H-NMR (CDCl_3_, 400MHz): δ 8.132-8.098 (2H, m, Ar-H); 7.580-7.560 (2H, d, *J* = 8.0Hz, Ar-H); 7.407-7.369 (2H, t, *J* = 7.2Hz, Ar-H); 7.197-7.159 (2H, t, *J* = 7.6Hz, Ar-H); 6.813-6.737 (4H, m, Ar-H); 4.569-4.536 (2H, t, *J* = 6.8Hz, CH_2_); 3.882-3.853 (2H, t, *J* = 5.6Hz, CH_2_); 3.695 (2H, s, CH_2_); 2.400-2.100 (2H, m, CH_2_); 1.446 (6H, s, CH_3_); 1.164 (3H, s, CH_3_). ^13^C-NMR (CDCl_3_, δ): 173.70 (C=O), 153.89(C aro), 148.49 (C aro), 139.91 (2×C aro), 125.62 (2×C aro), 122.05 (2×C aro), 121.38 (2×C aro), 121.25 (2×C aro), 120.22 (2×C aro), 118.72 (C aro), 114.99 (C aro), 109.06 (2×C aro), 79.19 (C(CH_3_)_2_), 64.81 (CH_2_), 60.83 (CH_2_), 52.17 (CH_2_), 28.14 (CH_2_), 24.90 (2×CH_3_), 13.89 (CH_3_). MS *m/z*: 432.13 (M+1), 454.20 (M+Na).

5f (Ethyl 2-(4-(3-(1H-benzo[d]imidazol-1-yl)propoxy)phenoxy)-2-methylpropanoate): colorless oil (33.23%), R_f_ = 0.65 (developing agent: dichloromethane/acetone = 10:1); ^1^H-NMR (CDCl_3_, 400MHz): δ 7.925 (1H, s, =CH); 7.818-7.802 (1H, q, *J* = 4.4Hz, Ar-H); 7.423-7.407 (1H, t, *J* = 7.2Hz, Ar-H); 7.291-7.265 (2H, m, Ar-H); 6.847-6.813 (2H, d, *J* = 8.8Hz, Ar-H); 6.761-6.739 (2H, d, *J* = 8.8Hz, Ar-H); 4.449-4.411 (2H, m, CH_2_); 4.269-4.215 (2H, m, CH_2_); 3.869-3.783 (2H, m, CH_2_); 2.308-2.294 (2H, m, CH_2_); 1.541 (6H, s, CH_3_); 1.300-1.255 (3H, m, CH_3_). ^13^C-NMR (CDCl_3_, δ): 174.26 (C=O), 154.08 (C aro), 149.38 (C aro), 143.72 (=CH), 143.18 (C aro),133.71 (C aro), 123.05 (C aro), 123.01 (C aro), 122.20 (2×C aro), 120.54 (C aro), 114.92 (2×C aro), 109.59 (C aro), 79.77 (C(CH_3_)_2_), 64.19 (CH_2_), 61.33 (CH_2_), 41.53 (CH_2_), 30.90 (CH_2_), 25.29 (2×CH_3_), 14.12 (CH_3_). MS *m/z*: 383.33 (M+1), 405.19 (M+Na), 765.87 (2M+1).

5g (Ethyl 2-methyl-2-(4-(3-phenylpropoxy)phenoxy)propanoate): colorless oil (29.24%), R_f_ = 0.52 (developing agent: dichloromethane); ^1^H-NMR (CDCl_3_, 400MHz): δ 7.247 (2H, m, Ar-H); 7.195 (3H, dd, *J* = 8.0Hz, Ar-H); 6.821 (2H, m, Ar-H); 6.762 (2H, m, Ar-H); 4.258 (2H, m, CH_2_); 3.901 (2H, t, *J* = 6.4Hz, CH_2_); 2.790 (2H, t, *J* = 7.2Hz, CH_2_); 2.075 (2H, m, CH_2_); 1.531 (6H, s, CH_3_); 1.273 (3H, t, *J* = 7.2Hz, CH_3_). ^13^C-NMR (CDCl_3_, δ): 174.31 (C=O), 154.72 (C aro), 148.96 (C aro), 141.58 (C aro), 128.52 (2×C aro), 128.42 (2×C aro), 125.93 (C aro), 121.63 (2×C aro), 114.91 (2×C aro), 79.69 (C(CH_3_)_2_), 67.34 (CH_2_), 61.28 (CH_2_), 32.20 (CH_2_), 30.94 (CH_2_), 25.33 (2×CH_3_), 14.14 (CH_3_). MS *m/z*: 343.07 (M+1), 706.93 (2M+Na).

5h (Ethyl 2-methyl-2-(3-(2-oxo-2-phenylethoxy)phenoxy)propanoate): achromatous oil (58.93%), R_f_ = 0.60 (developing agent: petroleum ether / EtOAc = 10:1); ^1^H-NMR (CDCl_3_, 400MHz): δ 8.093-7.974 (2H, m, Ar-H); 7.593 (1H, m, Ar-H); 7.512-7.474 (2H, t, *J* = 7.6Hz, Ar-H); 7.137-7.096 (1H, t, *J* = 8.0Hz, Ar-H); 6.597-6.571 (1H, m, Ar-H); 6.485-6.436 (2H, m, Ar-H); 5.217 (2H, s, CH_2_); 4.231-4.177 (2H, m, CH_2_); 1.581 (6H, s, CH_3_); 1.242-1.185 (3H, m, CH_3_). ^13^C-NMR (CDCl_3_, δ): 194.41 (C=O), 174.14 (C=O), 158.95 (C aro), 156.69 (C aro), 134.62 (C aro), 133.83 (C aro), 129.58 (C aro), 128.81 (2×C aro), 128.15 (2×C aro), 111.88 (C aro), 108.61 (C aro), 106.32 (C aro), 79.23 (C(CH_3_)_2_), 70.93 (CH_2_), 61.42 (CH_2_), 25.37 (2×CH_3_), 14.04 (CH_3_). MS *m/z*: 365.20 (M+Na), 706.87 (2M+Na).

5i (Ethyl 2-(3-(2-(1H-indol-1-yl)ethoxy)phenoxy)-2-methylpropanoate): achromatous oil (32.24%), R_f_ = 0.54 (developing agent: petroleum ether / EtOAc = 10:1); ^1^H-NMR (CDCl_3_, 400 MHz): δ 7.633-7.614 (1H, d, *J* = 7.6Hz, Ar-H); 7.402-7.382 (1H, d, *J* = 8.0Hz, Ar-H); 7.257-7.193 (4H, m, Ar-H); 7.125-7.055 (1H, m, =CH); 6.508-6.392 (3H, m, Ar-H and =CH); 4.511-4.483 (2H, t, *J* = 5.6Hz, CH_2_); 4.243-4.176 (4H, m, CH_2_); 1.569 (6H, s, CH_3_); 1.249-1.195 (3H, m, CH_3_). ^13^C-NMR (CDCl_3_, δ): 174.19 (C=O), 159.17 (C aro), 156.67 (C aro), 136.10 (C aro), 129.50 (2×C aro), 128.32 (=CH), 121.60 (C aro), 121.02 (C aro), 119.48 (C aro), 111.50 (C aro), 109.15 (C aro), 108.26 (C aro), 106.09 (C aro), 101.64 (=CH), 79.18 (C(CH_3_)_2_), 66.82 (CH_2_), 61.40 (CH_2_), 45.61 (CH_2_), 25.36 (2×CH_3_), 14.03 (CH_3_). MS *m/z*: 368.27 (M+1), 390.27 (M+Na), 756.93 (2M+Na).

5j (Ethyl 2-(3-(2-(9H-carbazol-9-yl)ethoxy)phenoxy)-2-methylpropanoate): white solid (13.70%), m.p. 57.6–60.1°C, R_f_ = 0.58 (developing agent: petroleum ether / EtOAc = 10:1); ^1^H-NMR (CDCl_3_, 400MHz): δ 8.136-8.097 (2H, m, Ar-H); 7.505-7.495 (4H, m, Ar-H); 7.241-7.217 (2H, m, Ar-H); 7.071-7.025 (1H, m, Ar-H); 6.450-6.347 (3H, m, Ar-H); 4.693-4.664 (2H, t, *J* = 6.0Hz, CH_2_); 4.302-4.272 (2H, t, *J* = 6.0Hz, CH_2_); 4.192-4.139 (2H, m, CH_2_); 1.562 (6H, s, CH_3_); 1.191-1.156 (3H, t, *J* = 7.2Hz, CH_3_). ^13^C-NMR (CDCl_3_, δ): 174.21 (C=O), 159.25 (C aro), 156.61 (C aro), 140.55 (2×C aro), 129.58 (C aro), 129.48 (C aro), 125.76 (2×C aro), 123.04 (C aro), 120.43 (2×C aro), 119.28 (2×C aro), 111.33 (C aro), 108.05 (2×C aro), 106.01 (C aro), 105.95 (C aro), 79.24 (C(CH_3_)_2_), 65.90 (CH_2_), 61.42 (CH_2_), 52.48 (CH_2_), 25.39 (2×CH_3_) , 14.03 (CH_3_). MS *m/z*: 440.20 (M+Na), 856.93 (2M+Na).

5k (Ethyl 2-(3-(3-(1H-indol-1-yl)propoxy)phenoxy)-2-methylpropanoate): achromatous oil (40.70%), R_f_ = 0.44 (developing agent: petroleum ether / EtOAc = 10:1); ^1^H-NMR (CDCl_3_, 400MHz): δ 7.626-7.607 (1H, d, *J* = 7.6Hz, Ar-H); 7.358-7.338 (1H, d, *J* = 8.0Hz, =CH); 7.251-7.061 (4H, m, Ar-H); 6.533-6.406 (4H, m, Ar-H and =CH); 4.366-4.333 (2H, t, *J* = 6.4Hz, CH_2_); 4.254-4.193 (2H, m, CH_2_); 3.840-3.749 (2H, m, CH_2_); 2.270-2.240 (2H, t, *J* = 6.0Hz, CH_2_); 1.595 (6H, s, CH_3_); 1.254-1.219 (3H, t, *J* = 6.8Hz, CH_3_). ^13^C-NMR (CDCl_3_, δ): 174.25 (C=O), 159.65 (C aro), 156.69 (C aro), 135.93 (C aro), 129.59 (C aro), 128.66 (C aro), 127.70 (C aro), 121.66 (C aro), 120.97 (C aro), 119.49 (C aro), 109.28 (C aro), 108.38 (C aro), 106.00 (C aro), 101.62 (C aro), 101.27 (C aro), 79.18 (C(CH_3_)_2_), 64.30 (CH_2_), 61.40 (CH_2_), 52.47 (CH_2_), 29.82 (CH_2_), 25.40 (2×CH_3_), 14.07 (CH_3_). MS *m/z*: 404.27 (M+Na).

5l (Ethyl 2-(3-(3-(9H-carbazol-9-yl)propoxy)phenoxy)-2-methylpropanoate): white solid (33.20%), m.p. 94.8-97.5°C, R_f_ = 0.64 (developing agent: dichloromethane); ^1^H-NMR (CDCl_3_, 400MHz): δ 8.092-8.073 (2H, d, *J* = 7.6Hz, Ar-H); 7.419-7.388 (4H, t, *J* = 6.4Hz, Ar-H); 7.244-7.104 (3H, m, Ar-H); 6.526-6.419 (3H, m, Ar-H); 4.543-4.511 (2H, t, *J* = 6.4Hz, CH_2_); 4.237-4.198 (2H, m, CH_2_); 3.874-3.846 (2H, t, *J* = 5.6Hz, CH_2_); 2.327-2.297 (2H, t, *J* = 6.0Hz, CH_2_); 1.587 (6H, s, CH_3_); 1.248-1.210 (3H, m, CH_3_). ^13^C-NMR (CDCl_3_, δ): 174.28 (C=O), 159.54 (C aro), 156.65 (C aro), 140.44 (2×C aro), 129.50 (C aro), 125.73 (2×C aro), 122.88 (2×C aro), 120.30 (2×C aro), 118.93 (2×C aro), 111.31 (C aro), 108.58 (2×C aro), 108.42 (C aro), 106.05 (C aro), 79.18 (C(CH_3_)_2_), 64.48 (CH_2_), 61.40 (CH_2_), 39.38 (CH_2_), 28.77 (CH_2_), 25.38 (2×CH_3_), 14.07 (CH_3_). MS *m/z*: 432.20 (M+1), 454.27 (M+Na).

5m (Ethyl 2-(3-(3-(1H-benzo[d]imidazol-1-yl)propoxy)phenoxy)-2-methylpropanoate): achromatous oil (40.12%), R_f_ = 0.58 (developing agent: dichloromethane / acetone = 10:1); ^1^H-NMR (CDCl_3_, 400MHz): δ 7.922 (1H, s, =CH); 7.825-7.803 (1H, m, Ar-H); 7.430-7.407(1H, m, Ar-H); 7.295-7.264 (2H, m, Ar-H); 7.140-7.099 (1H, t, *J* = 8.4Hz, Ar-H); 6.527-6.419 (3H, m, Ar-H); 4.445-4.412 (2H, t, *J* = 6.8Hz, CH_2_); 4.254-4.201 (2H, m, CH_2_); 3.883-3.855 (2H, t, *J* = 5.6Hz, CH_2_); 2.331-2.301 (2H, t, *J* = 6.0Hz, CH_2_); 1.601 (6H, s, CH_3_); 1.259-1.223 (3H, t, *J* = 7.2Hz, CH_3_). ^13^C-NMR (CDCl_3_, δ): 174.24 (C=O), 159.36 (C aro), 156.73 (C aro), 143.10 (=CH), 133.69 (C aro), 129.70 (C aro), 123.07 (C aro), 122.25 (C aro), 120.38 (C aro), 111.34 (C aro), 109.58 (C aro), 108.36 (C aro),108.22 (C aro), 105.88 (C aro),79.17 (C(CH_3_)_2_), 63.77 (CH_2_), 61.43 (CH_2_), 41.52 (CH_2_), 30.90 (CH_2_), 25.38 (2×CH_3_), 14.07 (CH_3_). MS *m/z*: 383.40 (M+1), 405.27 (M+Na), 787.20 (2M+Na).

5n (Ethyl 2-methyl-2-(3-(3-phenylpropoxy)phenoxy)propanoate): achromatous oil (12.66%), R_f_ = 0.55 (developing agent: petroleum ether / EtOAc = 10:1); ^1^H-NMR (CDCl_3_, 400MHz): δ 7.294-7.186 (6H, m, Ar-H); 7.116-7.075 (1H, t, *J* = 8.4Hz, Ar-H); 6.544-6.518 (1H, m, Ar-H); 6.439-6.393 (1H, m, Ar-H); 4.247-4.194 (2H, m, CH_2_); 3.919-3.888 (2H, t, *J* = 6.0Hz, CH_2_); 2.803-2.765 (2H, m, CH_2_); 2.093-2.055 (2H, m, CH_2_); 1.592 (6H, s, CH_3_); 1.255-1.219 (3H, t, *J* = 7.2Hz, CH_3_). ^13^C-NMR (CDCl_3_, δ): 174.29 (C=O), 160.00 (C aro), 156.65 (C aro), 141.51 (C aro), 129.39 (C aro), 128.54 (2×C aro), 128.49 (2×C aro), 125.92 (C aro), 110.98 (C aro), 108.55 (C aro), 105.95 (C aro), 79.13 (C(CH_3_)_2_), 66.91 (CH_2_), 61.38 (CH_2_), 32.16 (CH_2_), 30.80 (CH_2_), 25.41 (2×CH_3_); 14.07 (CH_3_). MS *m/z*: 365.13 (M+Na), 706.93 (2M+Na).

5o (Ethyl 2-methyl-2-(3-(pyridin-3-ylmethoxy)phenoxy)propanoate): achromatous oil (31.17%), R_f_ = 0.24 (developing agent: dichloromethane / acetone = 100:1); ^1^H-NMR (CDCl_3_, 400MHz): δ 8.673-8.581 (2H, m, Ar-H); 7.774 (1H, s, Ar-H); 7.359-7.327 (1H, m, Ar-H); 7.161-7.120 (1H, t, *J* = 8.4Hz, Ar-H); 6.627-6.601 (1H, m, Ar-H); 6.522-6.510 (1H, m, CH_2_); 6.463-6.436 (1H, d, *J* = 8.8Hz, CH_2_); 5.040 (2H, s, CH_2_); 4.255-4.202 (2H, m, CH_2_); 1.595 (6H, s, CH_3_); 1.263-1.228 (3H, t, *J* = 6.8Hz, CH_3_). ^13^C-NMR (CDCl_3_, δ): 174.20 (C=O), 159.23 (C aro), 156.72 (C aro), 149.07 (C aro), 148.61 (C aro), 135.57 (C aro), 132.72 (C aro), 129.63 (C aro), 123.67 (C aro), 111.62 (C aro), 108.58 (C aro), 106.17 (C aro), 79.20 (C(CH_3_)_2_), 67.45 (CH_2_), 61.46 (CH_2_), 25.38 (2×CH_3_), 14.07 (CH_3_). MS *m/z*: 338.20 (M+Na).

### Carboxylic acid compounds (6)

Method 1: esters (5, 10 mmol) were dissolved in the solution of THF-CH_3_OH-water (3:1:1, 100 ml). The reaction was beginning by adding 1M LiOH (30 ml, 30 mmol) dropwise to the mixture and stirred at room temperature for 1 h, followed by 6 M hydrochloric acid to acidify. The crude products were extracted with ethyl acetate, washed with water and saturated Na_2_CO_3_ solution and dehydrated with anhydrous Na_2_SO_4_. The solvent was removed by vacuum distillation to afford carboxylic acids (6a-l).

6a (2-(4-(2-(1H-indol-1-yl)ethoxy)phenoxy)-2-methylpropanoic acid): colorless oil (25.83%), R_f_ = 0.20 (developing agent: petroleum ether / EtOAc = 5:1); MS *m/z*: 338.13 (M-1), 340.20(M+1), 362.07(M+Na).

6b (2-(4-(2-(9H-carbazol-9-yl)ethoxy)phenoxy)-2-methylpropanoic acid): colorless oil (16.83%), R_f_ = 0.40 (developing agent: petroleum ether / EtOAc = 1:1); MS *m/z*: 388.20 (M-1), 412.13 (M+Na).

6c (2-(4-(3-(1H-indol-1-yl)propoxy)phenoxy)-2- methylpropanoic acid): colorless oil (36.75%), R_f_ = 0.36 (developing agent: petroleum ether / EtOAc = 1:1); ^1^H-NMR (CDCl_3_, 400MHz): δ 7.631-7.611 (1H, d, *J* = 8.0Hz, Ar-H); 7.358-7.338 (1H, d, *J* = 8.0Hz, =CH); 7.186-7.069 (3H, m, Ar-H); 6.920-6.897 (2H, m, Ar-H); 6.791-6.770 (2H, m, Ar-H); 6.477 (1H, s, =CH); 4.375-4.342 (2H, t, *J* = 6.8Hz, CH_2_); 3.838-3.810 (2H, t, *J* = 5.6Hz, CH_2_); 2.276-2.245 (2H, t, *J* = 6.0Hz, CH_2_); 1.549 (6H, s, CH_3_). ^13^C-NMR (CDCl_3_, δ): 175.96 (C=O), 155.37 (C aro), 147.25 (C aro), 135.92 (C aro), 128.63 (=CH), 128.05 (=CH), 123.23 (2×C aro), 121.53 (C aro), 121.00 (C aro), 119.36 (C aro), 115.04 (2×C aro), 109.29 (C aro), 101.30 (=CH), 80.66 (C(CH_3_)_2_), 64.70 (CH_2_), 42.72 (CH_2_), 29.87 (CH_2_), 24,75 (2×CH_3_). MS *m/z*: 352.13 (M-1).

6d (2-(4-(3-(9H-carbazol-9-yl)propoxy)phenoxy)-2- methylpropanoic acid): colorless oil (25.46%), R_f_ = 0.33 (developing agent: petroleum ether / EtOAc = 1:1); ^1^H-NMR (CDCl_3_, 400MHz): δ 8.099-8.080 (2H, d, *J* = 7.6Hz, Ar-H); 7.421-7.361 (4H, m, Ar-H); 7.258-7.189 (2H, m, Ar-H); 6.912-6.890 (2H, m, Ar-H); 6.787-6.764 (2H, m, Ar-H); 4.567-4.534 (2H, t, *J* = 6.8Hz, CH_2_); 3.873-3.845 (2H, t, *J* = 5.6Hz, CH_2_); 2.348-2.318 (2H, t, *J* = 6.0Hz, CH_2_); 1.536 (6H, s, CH_3_). ^13^C-NMR (CDCl_3_, δ): 176.06 (C=O), 155.13 (C aro), 147.56 (C aro), 140.42 (2×C aro), 125.71 (2×C aro), 122.98 (2×C aro), 122.86 (2×C aro), 120.32 (2×C aro), 118.94 (2×C aro), 115.02 (2×C aro), 108.59 (2×C aro), 97.48 (C(CH_3_)_2_), 64.87 (CH_2_), 55.06 (CH_2_), 39.34 (CH_2_), 30.92 (2×CH_3_). MS *m/z*: 402.13 (M-1), 805.07 (2M-1).

6e (2-(4-(3-(1H-benzo[d]imidazol-1-yl)propoxy)phenoxy)-2-methylpropanoic acid): colorless oil (31.25%), R_f_ = 0.35 (developing agent: dichloromethane / acetone = 1:1); ^1^H-NMR (CDCl_3_, 400MHz): δ 7.637-7.617 (1H, d, *J* = 8.0Hz, =CH); 7.403-7.383 (1H, d, *J* = 8.0Hz, Ar-H); 7.256-7.198 (3H, m, Ar-H); 6.875-6.843 (2H, m, Ar-H); 6.748-6.726 (2H, d, *J* = 8.0Hz, Ar-H); 4.515-4.487 (2H, t, *J* = 5.6Hz, CH_2_); 4.247-4.219 (2H, t, *J* = 5.6Hz, CH_2_); 3.486 (2H, s, CH_2_); 1.507 (6H, s, CH_3_). ^13^C-NMR (CDCl_3_, δ): 174.96 (C=O), 154.06 (C aro), 149.29 (C aro), 143.05 (=CH), 130.88 (C aro),123.15 (C aro), 122.38 (C aro), 121.74 (2×C aro), 120.29 (C aro), 114.93 (2×C aro), 109.64 (C aro), 106.74 (C aro), 79.78 (C(CH_3_)_2_), 64.21 (CH_2_), 52.39 (CH_2_), 30.88 (CH_2_), 25.38 (2×CH_3_). MS m/z: 353.13 (M-1).

6f (2-Methyl-2-(3-(2-oxo-2-phenylethoxy)phenoxy)propanoic acid): achromatous oil (10.48%), R_f_ = 0.20 (developing agent: petroleum ether / EtOAc = 1:1); ^1^H-NMR (CDCl_3_, 400MHz): δ 8.043-7.976 (2H, m, Ar-H); 7.636-7.599 (1H, t, *J* = 7.2Hz, Ar-H); 7.518-7.479 (2H, t, *J* = 8.0Hz, Ar-H); 7.173-7.132 (1H, t, *J* = 8.0Hz, Ar-H); 6.642-6.545 (3H, m, Ar-H); 5.233 (2H, s, CH_2_); 1.596 (6H, s, CH_3_). MS *m/z*: 313.07 (M-1), 337.13 (M+Na).

6g (2-(3-(2-(9H-carbazol-9-yl)ethoxy)phenoxy)-2-methylpropanoic acid): white solid (45.28%), m.p. 111.9-114.5°C, R_f_ = 0.28 (developing agent: petroleum ether/EtOAc = 1:1); MS *m/z*: 388.20 (M-1), 777.07 (2M-1).

6h (2-(3-(3-(1H-indol-1-yl)propoxy)phenoxy)-2-methylpropanoic acid): achromatous oil (19.48%), R_f_ = 0.24 (developing agent: dichloromethane / acetone = 10:1); ^1^H-NMR (CDCl_3_, 400MHz): δ 7.627-7.608 (1H, d, *J* = 7.6Hz, Ar-H); 7.353-7.253 (1H, m, =CH); 7.184-7.060 (4H, m, Ar-H); 6.604-6.466 (4H, m, Ar-H and =CH); 4.367-4.334 (2H, t, *J* = 6.4Hz, CH_2_); 3.845-3.817 (2H, t, *J* = 5.6Hz, CH_2_); 2.277-2.247 (2H, t, *J* = 6.0Hz, CH_2_); 1.598 (6H, s, CH_3_). ^13^C-NMR (CDCl_3_, δ): 176.79 (C=O), 159.65 (C aro), 155.64 (C aro), 135.93 (C aro), 129.71 (C aro), 128.65 (C aro), 128.05 (=CH), 121.53 (C aro), 120.99 (C aro), 119.34 (C aro), 112.69 (C aro), 109.41 (C aro), 109.28 (C aro), 107.38 (C aro), 101.30 (=CH), 79.75 (C(CH_3_)_2_), 64.42 (CH_2_), 42.72 (CH_2_), 30.89 (CH_2_), 25.09 (2×CH_3_). MS *m/z*: 352.13 (M-1), 354.20 (M+1), 376.13 (M+Na).

6i (2-(3-(3-(9H-carbazol-9-yl)propoxy)phenoxy)-2-methylpropanoic acid): achromatous oil (33.2%), R_f_ = 0.34 (developing agent: dichloromethane / acetone = 10:1); ^1^H-NMR (CDCl_3_, 400MHz): δ 8.092-8.073 (2H, d, *J* = 7.6Hz, Ar-H); 7.413-7.362 (4H, m, Ar-H); 7.251-7.119 (3H, m, Ar-H); 6.592-6.475 (3H, m, Ar-H); 4.548-4.515 (2H, t, *J* = 6.4Hz, CH_2_); 3.869-3.841 (2H, t, *J* = 5.6Hz, CH_2_); 2.337-2.307 (2H, t, *J* = 6.0Hz, CH_2_); 1.585 (6H, s, CH_3_). ^13^C-NMR (CDCl_3_, δ): 176.92 (C=O), 159.59 (C aro), 155.59 (C aro), 140.43 (2×C aro), 129.70 (C aro), 125.74 (2×C aro), 122.89 (2×C aro), 120.32 (2×C aro), 118.95 (2×C aro), 112.81 (C aro), 109.52 (C aro), 108.58 (2×C aro), 107.49 (C aro), 79.79 (C(CH_3_)_2_), 64.62 (CH_2_), 39.35 (CH_2_), 30.89 (CH_2_), 25.38 (2×CH_3_). MS *m/z*: 402.13 (M-1), 426.13 (M+Na).

6j (2-(3-(3-(1H-benzo[d]imidazol-1-yl)propoxy)phenoxy)-2-methylpropanoic acid): achromatous oil (16.43%), R_f_ = 0.38 (developing agent: dichloromethane); MS m/z: 353.20 (M-1), 355.13 (M+1).

6k (2-Methyl-2-(3-(3-phenylpropoxy)phenoxy)propanoic acid): achromatous oil form things (32.75%), R_f_ = 0.25 (developing agent: petroleum ether / EtOAc = 1:1); ^1^H-NMR (CDCl_3_, 400MHz): δ 7.305-7.257 (2H, m, Ar-H); 7.213-7.142 (4H, m, Ar-H); 6.644-6.503 (3H, m, Ar-H); 3.944-3.913 (2H, t, *J* = 6.4Hz, CH_2_); 2.816-2.778 (2H, t, *J* = 7.2Hz, CH_2_); 2.126-2.057 (2H, m, CH_2_); 1.599 (6H, s, CH_3_). ^13^C-NMR (CDCl_3_, δ): 176.07 (C=O), 160.03 (C aro), 155.38 (C aro), 141.43 (C aro), 129.62 (C aro), 128.49 (2×C aro), 128.41 (2×C aro), 125.94 (C aro), 112.60 (C aro), 109.63 (C aro), 107.59 (C aro), 79.93 (C(CH_3_)_2_), 67.03 (CH_2_), 32.13 (CH_2_), 30.88 (CH_2_), 25.10 (2×CH_3_). MS *m/z*: 313.07 (M-1), 337.07 (M+Na).

6l (2-Methyl-2-(3-(pyridin-3-ylmethoxy)phenoxy)propanoic acid): achromatous oil (14.63%), R_f_ = 0.42 (developing agent: petroleum ether / EtOAc = 1:1); MS m/z: 286.07 (M-1), 288.20 (M+1), 597.00 (2M+Na).

Method 2: esters (5, 10 mmol) were dissolved in CH_3_OH (25 ml), adding 1 M NaOH (10 ml, 10 mmol) under the stirring condition in ice bath. With the solution up to the room temperature, the reaction was heated to reflux at 50°C for 24 h under stirring condition, followed by 1 M hydrochloric acid (10 ml, 10 mmol) for 5 h. The crude products were obtained after filtration and the solvent removed by vacuum distillation. The purification was washed for three times with CH_3_OH-water (1:1) to afford carboxylic acids (6m-o).

6m (2-Methyl-2-(4-(3-phenylpropoxy)phenoxy)propanoic acid): white solid (31.85%), m.p. 112.1-114.2°C, R_f_ = 0.75 (developing agent: dichloromethane / acetone = 1:1); ^1^H-NMR (CDCl_3_, 400MHz): δ 7.288-7.178 (5H, m, Ar-H); 6.906 (2H, d, *J* = 8.8Hz, Ar-H); 6.802 (2H, d, *J* = 9.2Hz, Ar-H); 4.758 (1H, s, OH); 3.924 (2H, t, *J* = 6.0Hz, CH_2_); 2.803 (2H, t, *J* = 7.6Hz, CH_2_); 2.127-2.058 (2H, m, CH_2_); 1.535 (6H, s, CH_3_). ^13^C-NMR (CDCl_3_, δ): 176.72 (C=O), 155.68 (C aro), 147.13 (C aro), 141.48 (C aro), 128.51 (2×C aro), 128.43 (2×C aro), 125.96 (C aro), 121.69 (2×C aro), 115.0 (2×C aro), 80.53 (C(CH_3_)_2_), 67.32 (CH_2_), 32.15 (CH_2_), 30.85 (CH_2_), 24.79 (2×CH_3_). MS *m/z*: 313.20 (M-1).

6n (2-Methyl-2-(4-(2-oxo-2-phenylethoxy)pheno xy)propanoic acid): colorless oil (32.41%), R_f_ = 0.42 (developing agent: petroleum ether / EtOAc = 1:1); ^1^H-NMR (CDCl_3_, 400MHz): δ 8.081-7.982 (2H, m, Ar-H); 7.641-7.604 (1H, d, *J* = 7.2Hz, Ar-H); 7.523-7.468 (2H, m, Ar-H); 6.932-6.848 (4H, m, Ar-H); 5.243 (2H, s, CH_2_); 1.561 (6H, s, CH_3_). MS *m/z*: 313.20 (M-1); 337.13 (M+Na).

6o (2-(3-(2-(1H-indol-1-yl)ethoxy)phenoxy)-2-methylpropanoic acid): achromatous oil (36.47%), R_f_ = 0.34 (developing agent: dichloromethane); ^1^H-NMR (CDCl_3_, 400MHz): δ 7.636-7.617 (1H, d, *J* = 7.6Hz, Ar-H); 7.401-7.380 (1H, d, *J* = 8.4Hz, =CH); 7.255-7.189 (2H, m, Ar-H); 7.135-7.090 (2H, m, Ar-H); 6.551-6.438 (4H, m, Ar-H and =CH); 4.507-4.479 (2H, t, *J* = 5.6Hz, CH_2_); 4.242-4.214 (2H, t, *J* = 5.6Hz, CH_2_); 1.570 (6H, s, CH_3_). ^13^C-NMR (CDCl_3_, δ): 173.19 (C=O), 159.25 (C aro), 136.04 (C aro), 129.76 (C aro), 128.68 (C aro), 128.36 (=CH), 121.64 (C aro), 121.07 (2×C aro), 119.53 (C aro), 109.57 (C aro), 109.17 (2×C aro), 107.60 (C aro), 101.69 (=CH), 79.18 (C(CH_3_)_2_), 66.95 (CH_2_), 45.62 (CH_2_), 25.36 (2×CH_3_). MS *m/z*: 338.20 (M-1), 677.00 (2M-1).

### *Para*-/*meta*- monosubstituted intermediates (8)

Hydroquinone / resorcinol 1 (2.2 g, 20 mmol) was dissolved in the solution of acetone (50 ml), followed by K_2_CO_3_ (2.76 g, 20 mmol) and NaI (catalytic amounts). Then a mixed solution of chloride acetonitrile 7 (0.755 g, 10 mmol) and acetone (50 ml) was dripped slowly to the mixture above. The reaction was heated to reflux at 65°C for 16 h under the stirring condition. The process of the reaction was detected through TLC method. After the reaction, crude products were obtained through the course of filtration and vacuum distillation to remove the solvent. The intermediates 8 were purified by flash column chromatography with petroleum ether/EtOAc as the mobile phase.

8a (2-(4-Hydroxyphenoxy)acetonitrile): achromatous oil (80.62%), R_f_ = 0.38 (developing agent: dichloromethane / methanol = 50:1); ^1^H-NMR (CDCl_3_, 400MHz): δ 6.914-6.882 (2H, m, Ar-H); 6.828-6.787 (2H, m, Ar-H); 4.702 (2H, s, CH_2_). ^13^C-NMR (CDCl_3_, 101MHz): 151.54 (C aro), 150.80 (C aro), 117.02 (2×C aro), 116.41 (2×C aro), 115.33 (C≡N), 55.04 (CH_2_). MS *m/z*: 147.93 (M-1), 296.80 (2M-1).

8b (2-(3-Hydroxyphenoxy)acetonitrile): white solid (84.7%), R_f_ = 0.46 (developing agent: dichloromethane / methanol = 50:1); ^1^H-NMR (CDCl_3_, 400MHz): δ 7.262 (1H, s, Ar-H); 7.217-7.176 (1H, t, *J* = 8.2 Hz, Ar-H); 6.583-6.539 (2H, t, *J* = 8.8 Hz, Ar-H); 4.743 (2H, s, CH_2_). ^13^C-NMR (CDCl_3_, 101MHz): 158.69 (C aro), 157.49 (C aro), 130.15 (C aro), 116.70 (C≡N), 109.61 (C aro), 105.17 (C aro), 102.15 (C aro), 53.26 (CH_2_).

### Nitrile intermediates (9)

Monosubstituted intermediates 8 (1.49 g, 10 mmol), halogenated organic compounds 4 (10 mmol), K_2_CO_3_ (3.32 g, 24 mmol) and NaI (catalytic amounts) were added to 50 ml acetone. The solution was heated to reflux at 65°C for 12 h under stirring condition. The process of the reaction was detected through TLC method. After the reaction, the final products were obtained through the course of filtration and vacuum distillation to remove the solvent. Nitrile intermediates (9) were purified by flash column chromatography with petroleum ether/EtOAc as the mobile phase.

9a (2-(4-(2-(1H-indol-1-yl)ethoxy)phenoxy)aceto nitrile): achromatous oil (20.90%), R_f_ = 0.60 (developing agent: petroleum ether / EtOAc = 3:1); ^1^H-NMR (CDCl_3_, 400 MHz): δ 7.643-7.624 (1H, d, *J* = 7.6 Hz, Ar-H); 7.405-7.385 (1H, d, *J* = 8.0 Hz, Ar-H); 7.248-7.188 (2H, m, Ar-H); 7.115(1H, s, Ar-H), 6.888-6.852 (2H, m, Ar-H); 6.797-6.762 (2H, m, Ar-H); 6.518-6.504 (1H, t, *J* = 2.8 Hz, Ar-H); 4.654 (2H, s, CH_2_); 4.511-4.497 (2H, t, *J* = 2.8 Hz, CH_2_); 4.239-4.226 (2H, t, *J* = 2.6 Hz, CH_2_). ^13^C-NMR (CDCl_3_, 101 MHz): δ 154.41 (C aro), 151.03 (C aro), 137.05 (C aro), 128.67 (C aro), 128.37 (=C-N), 127.03 (C aro), 121.63 (C aro), 121.48 (C aro), 119.52 (C aro), 115.78 (C≡N), 115.33 (C aro), 115.30 (C aro),109.19 (C aro), 109.02 (C aro), 101.66 (=CH), 67.53 (CH_2_), 54.78 (CH_2_), 45.68 (CH_2_). MS *m/z*: 293.20(M+1), 585.27(2M+1).

9b (2-(4-(2-(9H-carbazol-9-yl)ethoxy)phenoxy)acetonitrile): white solid (12.63%), m.p. 99.8-102.1°C, R_f_ = 0.62 (developing agent: petroleum ether / EtOAc = 3:1); ^1^H-NMR (DMSO-d_6_, 400 MHz): δ 8.154-8.135 (2H, d, *J* = 7.6 Hz, Ar-H); 7.686-7.665 (2H, d, *J* = 8.4 Hz, Ar-H); 7.504-7.439 (2H, m, Ar-H); 7.224-7.187 (2H, t, *J* = 7.4 Hz, Ar-H); 6.824-6.696 (4H, m, Ar-H); 4.785-4.759 (2H, t, *J* = 5.2 Hz, CH_2_); 4.323-4.287 (4H, t, *J* = 7.2 Hz, CH_2_). ^13^C-NMR (DMSO-d_6_, 101MHz): δ 152.55 (C aro), 151.89 (C aro), 140.21 (2×C aro), 125.60 (2×C aro), 122.09 (2×C aro), 120.11 (2×C aro), 118.84 (2×C aro), 115.56 (C aro, C≡N), 115.09 (2×C aro), 114.81 (C aro), 109.58 (2×C aro), 67.29 (CH_2_), 66.81 (CH_2_), 42.13 (CH_2_). MS *m/z*: 341.00 (M-1).

9c (2-(4-(3-(1H-indol-1-yl)propoxy)phenoxy)acetonitrile): achromatous oil (36.75%), R_f_ = 0.46 (developing agent: petroleum ether / EtOAc = 3:1); ^1^H-NMR (CDCl_3_, 400 MHz): δ 7.638-7.618 (1H, d, *J* = 8.0 Hz, Ar-H); 7.369-7.348 (1H, d, *J* = 8.4 Hz, Ar-H); 7.201-7.160 (1H, m, Ar-H); 7.115-7.077 (2H, m, Ar-H); 6.945-6.866 (2H, m, Ar-H); 6.857-6.835 (2H, m, Ar-H); 6.481 (1H, s, Ar-H); 4.710 (2H, s, CH_2_); 4.390-4.357 (2H, t, *J* = 6.6 Hz, CH_2_); 3.845-3.817 (2H, t, *J* = 5.6 Hz, CH_2_); 2.305-2.244 (2H, m, CH_2_). ^13^C-NMR (CDCl_3_, 101MHz): δ 154.80 (C aro), 150.84 (C aro), 136.88 (C aro), 128.12 (C aro), 126.90 (=CH), 121.54 (C aro), 120.92 (C aro), 119.36 (C aro), 118.07 (C aro), 116.77 (C aro), 115.63 (C≡N), 115.61 (C aro), 115.34 (C aro), 109.30 (=CH), 101.30 (C aro), 64.94 (CH_2_), 54.90 (CH_2_), 42.70 (CH_2_), 30.95 (CH_2_). MS *m/z*: 329.13 (M+Na).

9d (2-(4-(3-(9H-carbazol-9-yl)propoxy)phenoxy)acetonitrile): achromatous oil (70.60%), R_f_ = 0.50 (developing agent: petroleum ether / EtOAc = 3:1); ^1^H-NMR (CDCl_3_,400 MHz): δ 8.105-8.086 (2H, d, J = 7.6 Hz, Ar-H); 7.432-7.378 (4H, m, Ar-H); 7.258-7.196 (2H, m, Ar-H); 6.935-6.903 (2H, m, Ar-H); 6.854-6.823 (2H, m, Ar-H); 4.709 (2H, s, CH_2_); 4.573-4.541 (2H, t, *J* = 6.4 Hz, CH_2_); 3.880-3.852 (2H, t, *J* = 5.6 Hz, CH_2_); 2.370-2.325 (2H, m, CH_2_). ^13^C-NMR (CDCl_3_, 101MHz): δ 154.66 (C aro), 150.82 (C aro), 140.43 (2×C aro), 125.75 (2×C aro), 122.87 (2×C aro), 120.35 (2×C aro), 118.97 (2×C aro), 116.73 (2×C aro), 115.65 (2×C aro), 115.34 (C≡N), 108.57 (2×C aro), 65.02 (CH_2_), 54.89 (CH_2_), 39.34 (CH_2_), 30.96 (CH_2_). MS *m/z*: 379 (M+Na), 712.47 (2M).

9e (2-(4-(3-Phenylpropoxy)phenoxy)acetonitrile): achromatous oil (15.84%), R_f_ = 0.52 (developing agent: petroleum ether / EtOAc = 3:1); ^1^H-NMR (CDCl_3_, 400 MHz): δ 7.307-7.177 (5H, m, Ar-H); 6.949-6.838 (4H, m, Ar-H); 4.697 (2H, s, CH_2_); 3.938-3.906 (2H, t, *J* = 6.4Hz, CH_2_); 2.822-2.784 (2H, t, *J* = 7.6Hz, CH_2_); 2.166-2.041(2H, m, CH_2_). ^13^C-NMR (CDCl_3_, 101MHz): 155.10 (C aro), 150.66 (C aro), 141.46 (C aro), 128.52 (2×C aro), 128.44 (2×C aro), 125.97 (C aro), 116.74 (2×C aro), 115.64 (2×C aro), 115.41 (C≡N), 67.43 (CH_2_), 54.96 (CH_2_), 32.13 (CH_2_ ), 30.84(CH_2_). MS *m/z*: 290.07 (M+Na), 535.00 (2M+1), 556.93(2M+Na).

9f (2-(4-(Pyridin-3-ylmethoxy)phenoxy)aceto nitrile): achromatous oil (32.10%), R_f_ = 0.34 (developing agent: petroleum ether / EtOAc = 3:1); ^1^H-NMR (CDCl_3_, 400MHz): δ 8.680 (1H, s, Ar-H); 8.599-8.587 (1H, d, *J* = 4.8 Hz, Ar-H); 7.790-7.771 (1H, d, *J* = 7.6 Hz, Ar-H); 7.355-7.324 (1H, dd, *J* = 7.6 Hz, Ar-H); 6.977 (4H, s, Ar-H); 5.052 (2H, s, CH_2_); 4.720 (2H, s, CH_2_). ^13^C-NMR (CDCl_3_, 101MHz): δ 154.31 (C aro), 151.14 (C aro), 149.37 (C aro), 148.85 (C aro), 135.41 (C aro), 132.47 (C aro), 123.59 (C aro), 116.75 (2×C aro), 116.05 (2×C aro), 115.29 (C≡N), 68.10 (CH_2_), 54.78 (CH_2_). MS *m/z*: 263.13 (M+Na), 479.07 (2M-1), 480.87 (2M+1), 502.93 (2M+Na).

9g (2-(3-(2-(1H-indol-1-yl)ethoxy)phenoxy)aceto nitrile): corlorless oil (18.21%), R_f_ = 0.43 (developing agent: petroleum ether / EtOAc = 3:1); ^1^H-NMR (CDCl_3_, 400 MHz): δ 7.643-7.624 (1H, d, *J* = 7.6 Hz, Ar-H); 7.412-7.391 (1H, d, *J* = 8.4 Hz, Ar-H); 7.253-7.102 (4H, m, Ar-H); 6.569-6.507 (3H, m, Ar-H); 6.384-6.372 (1H, t, *J* = 2.4 Hz, Ar-H); 4.614 (2H, s, CH_2_); 4.523-4.496 (2H, t, *J* = 5.4 Hz, CH_2_); 4.276-4.248 (2H, t, *J* = 5.6 Hz, CH_2_). ^13^C-NMR (CDCl_3_, 101MHz): δ 159.70 (C aro), 157.67 (C aro), 136.03 (C aro), 130.42 (C aro), 128.82 (C aro), 128.40 (=C-N), 121.72 (C aro), 121.11 (C aro), 119.60 (C aro), 115.02 (C≡N), 109.22 (C aro), 109.19 (C aro), 107.39 (C aro), 102.22 (C aro), 101.76 ( = CH), 67.09 (CH_2_), 53.51 (CH_2_), 45.68 (CH_2_). MS *m/z*: 293.13 (M+1), 315.20 (M+Na).

9h (2-(3-(2-(9H-carbazol-9-yl)ethoxy)phenoxy)acetonitrile): corlorless oil (12.66%), R_f_ = 0.60 (developing agent: petroleum ether / EtOAc = 3:1); 1H-NMR (CDCl_3_, 400 MHz): δ 8.099-8.079 (2H, d, *J* = 8.0Hz, Ar-H); 7.501-7.431 (4H, m, Ar-H); 7.266-7.227 (2H, m, Ar-H); 7.159-7.117 (1H, t, *J* = 8.4 Hz, Ar-H); 6.520-6.482 (2H, m, Ar-H); 6.363-6.352 (1H, t, *J* = 2.2 Hz, Ar-H); 4.708-4.679 (2H, t, *J* = 5.8 Hz, CH2); 4.626-4.595 (2H, t, *J* = 6.2 Hz, CH2); 4.338-4. 308 (2H, t, *J* = 6.0 Hz, CH2). 13C-NMR (CDCl3, 101MHz): δ 159.68 (C aro), 157.61 (C aro), 140.51 (C aro), 140.14 (C aro),130.34 (C aro), 125.94 (C aro), 125.81 (C aro), 123.12 (C aro), 123.04 (C aro), 120.51 (C aro), 120.30 (C aro), 119.53 (C aro), 119.36 (C aro), 115.01 (C≡N), 109.09 (C aro), 108.76 (C aro), 108.49 (C aro), 107.19 (C aro), 102.19 (C aro), 66.16 (CH2), 53.49 (CH2), 44.71 (CH2). MS *m/z*: 343.07 (M+1).

9i (2-(3-(3-(1H-indol-1-yl)propoxy)phenoxy)acetonitrile): corlorless oil (19.10%), R_f_ = 0.52 (developing agent: petroleum ether / EtOAc = 3:1); ^1^H-NMR (CDCl_3_, 400 MHz): δ 7.636-7.617 (1H, d, *J* = 7.6 Hz, Ar-H); 7.367-7.346 (1H, d, *J* = 8.4 Hz, =CH); 7.182-7.164 (2H, d, *J* = 7.2 Hz, Ar-H); 7.114-7.094 (2H, d, *J* = 8.0 Hz, Ar-H); 6.609-6.559 (2H, m, Ar-H); 6.495-6.477 (2H, m, Ar-H and =CH); 4.714 (2H, s, CH_2_); 4.384-4.351 (2H, t, *J* = 6.6 Hz, CH_2_); 3.872-3.844 (2H, t, *J* = 5.6 Hz, CH_2_); 2.297-2.267 (2H, t, *J* = 6.0 Hz, CH_2_). ^13^C-NMR (CDCl_3_, 101MHz): δ 154.86 (C aro), 152.47 (C aro), 131.66 (C aro), 130.66 (C aro), 125.17 (C aro), 125.13 (=CH), 123.41 (C aro), 119.24 (C aro), 118.96 (C aro), 115.99 (C≡N), 109.84 (C aro), 104.04 (C aro), 103.97 (C aro), 101.85 (=CH), 101.72 (C aro), 59.46 (CH_2_), 59.28 (CH_2_), 48.33 (CH_2_), 29.37 (CH_2_). MS *m/z*: 307.27 (M+1).

9j (2-(3-(3-(9H-carbazol-9-yl)propoxy)phenoxy)acetonitrile): corlorless oil (32.11%), R_f_ = 0.54 (developing agent: petroleum ether / EtOAc = 3:1); ^1^H-NMR (CDCl_3_, 400 MHz): δ 8.090-8.071 (2H, d, *J* = 7.6 Hz, Ar-H); 7.401-7.369 (4H, t, *J* = 6.4 Hz, Ar-H); 7.233-7.168 (3H, m, Ar-H); 6.561-6.528 (2H, m, Ar-H); 6.458 (1H, s, Ar-H); 4.667 (2H, s, CH_2_); 4.537-4.505 (2H, t, *J* = 6.4 Hz, CH_2_); 3.872-3.845 (2H, t, *J* = 5.4 Hz, CH_2_); 2.330-2.252 (2H, m, CH_2_). ^13^C-NMR (CDCl_3_, 101MHz): δ 154.78 (C aro), 152.46 (C aro), 135.19 (3×C aro), 125.15 (2×C aro), 120.59 (4×C aro), 120.54 (2×C aro), 117.66 (C≡N), 109.87 (2×C aro), 103.40 (C aro), 103.36 (C aro), 101.70 (C aro), 59.53 (CH_2_), 55.14 (CH_2_), 34.11 (CH_2_), 25.66 (CH_2_).

9k (2-(3-(3-Phenylpropoxy)phenoxy)acetonitrile): corlorless oil (32.40%), R_f_ = 0.55 (developing agent: petroleum ether / EtOAc = 3:1); ^1^H-NMR (CDCl_3_, 400MHz): δ 7.312-7.249 (3H, m, Ar-H); 7.228-7.182 (3H, m, Ar-H); 6.633-6.607 (1H, m, Ar-H); 6.571-6.512 (2H, m, Ar-H); 4.741 (2H, s, CH_2_); 3.966-3.935 (2H, t, *J* = 6.2 Hz, CH_2_); 2.828-2.790 (2H, t, *J* = 7.6 Hz, CH_2_); 2.140-2.071 (2H, m, CH_2_). ^13^C-NMR (CDCl_3_, 101MHz): 160.46 (C aro), 157.71 (C aro), 141.38 (C aro), 130.31 (C aro), 128.52 (2×C aro), 128.45 (2×C aro), 125.98 (C aro), 115.13 (C≡N), 109.19 (C aro), 106.60 (C aro), 102.24 (C aro), 67.06 (CH_2_), 53.58 (CH_2_), 32.11 (CH_2_ ), 30.92 (CH_2_).

9l (2-(3-(Pyridin-3-ylmethoxy)phenoxy)aceto nitrile): corlorless oil (19.51%), R_f_ = 0.45 (developing agent: petroleum ether / EtOAc = 1:1); ^1^H-NMR (CDCl_3_, 400MHz): δ 8.687 (1H, s, Ar-H); 8.597 (1H, s, Ar-H); 7.799-7.779 (1H, d, *J* = 8 Hz, Ar-H); 7.363-7.335 (1H, m, Ar-H); 7.292-7.249 (1H, m, Ar-H); 6.720-6.699 (1H, d, *J* = 8.4 Hz, Ar-H); 6.615-6.610 (2H, d, *J* = 2.0 Hz, Ar-H); 5.076 (2H, s, CH_2_); 4.757 (2H, s, CH_2_). ^13^C-NMR (CDCl_3_, 101MHz): δ 159.74 (C aro), 157.76 (C aro), 149.43 (C aro), 148.84 (C aro), 135.43 (C aro), 132.22 (C aro), 130.53 (C aro),123.60 (C aro), 115.01 (C≡N),109.34 (C aro), 107.39 (C aro), 102.73 (C aro), 67.69 (CH_2_), 53.61 (CH_2_). MS *m/z*: 241.13 (M+1), 263.07 (M+Na), 480.60 (M+1), 502.73 (2M+Na).

### Tetrazole compounds (10)

The mixture of toluene (24 ml), nitrile intermediates 9 (10 mmol), triethylamine hydrochloride (2.48 g, 18 mmol) and sodium azide (1.12 g, 16 mmol) was heated at 120°C for 48 h. The process of the reaction was detected through TLC method. After the reaction, on cooling to room temperature, ice-water was added to the solution. Solid was precipitated after the pH of water layer adjusted to 1.0 with diluted hydrochloric acid. The solid products were further purified by flash column chromatography with dichloromethane/methanol as the mobile phase.

10a (1-(2-(4-((2H-tetrazol-5-yl)methoxy)phenoxy)ethyl)-1H-indole): white solid (61.03%), m.p. 132.3-136.1°C, R_f_ = 0.39 (developing agent: dichloromethane / methanol = 10:1); ^1^H-NMR (DMSO-d_6_, 400 MHz): δ 7.551-7.529 (2H, m, Ar-H); 7.419-7.411 (1H, d, *J* = 3.2 Hz, Ar-H); 7.137-7.113(1H, m, Ar-H); 7.034-7.013 (1H, dd, *J* = 3.8 Hz, Ar-H); 6.964-6.942 (2H, dd, *J* = 3.4 Hz, Ar-H); 6.847-6.824 (2H, dd, *J* = 3.4 Hz, Ar-H); 6.433-6.431 (1H, d, *J* = 0.8 Hz, Ar-H); 5.378 (2H, d, CH_2_); 4.552-4.426 (2H, t, *J* = 5.2 Hz, CH_2_); 4.246-4.219 (2H, t, *J* = 5.4 Hz, CH_2_). ^13^C-NMR (DMSO-d_6_, 101MHz): δ 158.16 (C=N), 156.81 (C aro), 141.11 (C aro), 134.26 (C aro), 133.31 (C aro), 126.22 (=CH-N), 125.55 (C aro), 124.20 (C aro), 121.19 (C aro), 120.60 (2×C aro), 115.16 (2×C aro), 105.92 (=CH, C aro), 72.60 (CH_2_), 65.11 (CH_2_), 50.28 (CH_2_). MS *m/z*: 334.13(M-1); 335.13(M); 336.13(M+1).

10b (9-(2-(4-((2H-tetrazol-5-yl)methoxy)phenoxy)ethyl)-9H-carbazole): white solid (30.70%), m.p. 131.4-139.8°C, R_f_ = 0.42 (developing agent: dichloromethane/methanol = 10:1); ^1^H-NMR (DMSO-d_6_, 400 MHz): δ 8.170-8.128 (2H, t, *J* = 8.4 Hz, Ar-H); 7.684-7.663 (2H, d, *J* = 8.4 Hz, Ar-H); 7.478-7.440 (2H, t, *J* = 7.6 Hz, Ar-H); 7.226-7.189 (2H, t, *J* = 7.4 Hz, Ar-H); 6.929-6.897 (2H, t, *J* = 6.4 Hz, Ar-H); 6.764-6.741 (2H, d, *J* = 9.2 Hz, Ar-H); 5.357 (2H, s, CH_2_); 4.786-4.760 (2H, t, *J* = 5.2 Hz, CH_2_); 4.314-4.288 (2H, t, *J* = 5.2 Hz, CH_2_). ^13^C-NMR (DMSO-d_6_, 101MHz): δ 152.94 (C=N, C aro), 151.48 (C aro), 140.21 (2×C aro), 125.60 (2×C aro), 122.09 (2×C aro), 120.11 (2×C aro), 118.85 (2×C aro), 115.91 (2×C aro), 115.68 (C aro), 114.19 (C aro), 109.58 (2×C aro), 66.80 (CH_2_), 59.83 (CH_2_), 54.87 (CH_2_). MS *m/z*: 384.07 (M-1); 385.07(M); 386.14(M+1); 407.73(M+Na).

10c (1-(3-(4-((2H-tetrazol-5-yl)methoxy)phenoxy)propyl)-1H-indole): yellowish solid (57.77%), m.p. 148.5-153.7°C, R_f_ = 0.42 (developing agent: dichloromethane / methanol = 30:1); ^1^H-NMR (DMSO-d_6_, 400MHz): δ 7.543-7.523 (1H, d, *J* = 8.0 Hz, Ar-H); 7.469-7.449 (1H, d, *J* = 8.0 Hz, Ar-H); 7.345-7.338 (1H, d, *J* = 2.8 Hz, Ar-H); 7.110-7.072 (1H, m, Ar-H); 7.016-6.897 (3H, m, Ar-H); 6.888-6.856 (2H, m, Ar-H); 6.423-6.416 (1H, d, *J* = 2.8Hz, Ar-H); 5.402 (2H, s, CH_2_); 4.356-4.322 (2H, t, *J* = 6.8 Hz, CH_2_); 3.842-3.812 (2H, t, *J* = 6.0 Hz, CH_2_); 2.199-2.135 (2H, m, CH_2_). ^13^C-NMR (DMSO-d_6_, 101MHz): δ 153.23 (C aro, C=N), 151.45 (C aro), 135.61 (C aro), 128.60 (C aro), 128.05 (=CH), 120.97 (C aro), 120.39 (C aro), 118.87 (C aro), 115.94 (2×C aro), 115.38 (2×C aro), 109.62 (C aro), 100.59 (=CH), 64.94 (CH_2_), 59.87 (CH_2_), 42.18 (CH_2_), 30.66 (CH_2_). MS *m/z*: 348.00 (M-1), 349.20 (M), 350.13 (M+1).

10d (9-(3-(4-((2H-tetrazol-5-yl)methoxy)phenoxy)propyl)-9H-carbazole): white solid (66.18%), m.p. 168.2-171.3°C, R_f_ = 0.30 (developing agent: dichloromethane/methanol = 30:1); ^1^H-NMR (DMSO-d_6_, 400MHz): δ 8.157-8.137 (2H, d, *J* = 8.0 Hz, Ar-H); 7.592-7.572 (2H, d, *J* = 8.0 Hz, Ar-H); 7.413-7.375 (2H, dd, *J* = 8.0 Hz Ar-H); 7.201-7.164 (2H, t, *J* = 7.4 Hz, Ar-H); 6.984-6.961 (2H, d, *J* = 8.4 Hz, Ar-H); 6.872-6.850 (2H, d, *J* = 8.0 Hz, Ar-H); 5.401 (2H, s, CH_2_); 4.577-4.544 (2H, t, *J* = 6.6 Hz, CH_2_); 3.880-3.852 (2H, t, *J* = 5.6 Hz, CH_2_); 2.245-2.171 (2H, m, CH_2_). ^13^C-NMR (DMSO-d_6_, 101MHz): δ 152.06 (C aro, C=N), 151.12 (C aro), 139.92 (2×C aro), 125.65 (2×C aro), 122.04 (2×C aro), 120.24 (2×C aro), 118.73 (2×C aro), 115.91 (2×C aro), 115.38 (2×C aro), 109.07 (2×C aro), 64.93 (2×CH_2_), 59.89 (CH_2_), 30.66 (CH_2_). MS *m/z*: 398.13 (M-1), 797.07 (2M-1).

10e (5-((4-(3-Phenylpropoxy)phenoxy)methyl)-2H-tetrazole): white solid (64.52%), m.p. 142.5-147.8°C, R_f_ = 0.37 (developing agent: dichloromethane / methanol = 30:1); ^1^H-NMR (DMSO-d_6_, 400MHz): δ 7.302-7.159 (5H, m, Ar-H); 6.966-6.943 (2H, d, *J* = 9.2 Hz, Ar-H); 6.841-6.818 (2H, d, *J* = 9.2 Hz, Ar-H); 5.052 (2H, s, CH_2_); 3.902-3.870 (2H, t, *J* = 6.4 Hz, CH_2_); 2.744- 2.670 (2H, m, CH_2_); 2.012-1.942 (2H, m, CH_2_). ^13^C-NMR (DMSO-d_6_, 101MHz): 152.67 (C aro, C=N), 152.48 (C aro), 141.44 (C aro), 128.32 (2×C aro), 128.29 (2×C aro), 125.76 (C aro), 115.44 (2×C aro), 115.18 (2×C aro), 67.07 (CH_2_), 61.95 (CH_2_), 31.48 (CH_2_ ), 30.66 (CH_2_). MS m/z: 309.13 (M-1).

10f (3-((4-((2H-tetrazol-5-yl)methoxy)phenoxy)methyl)pyridine): yellowish solid (97.67%), m.p. 190.7-192.2°C, R_f_ = 0.30 (developing agent: dichloromethane / methanol = 30:1); ^1^H-NMR (DMSO-d_6_, 400MHz): δ 8.656 (1H, s, Ar-H); 8.549-8.533 (1H, dd, *J* = 4.8Hz, Ar-H); 7.867-7.847 (1H, d, *J* = 8.0Hz, Ar-H); 7.440-7.409 (1H, dd, *J* = 7.6Hz, Ar-H); 7.026-6.976 (4H, m, Ar-H); 5.415 (2H, s, CH_2_); 5.097 (2H, s, CH_2_). ^13^C-NMR (DMSO-d_6_, 101MHz): 152.88 (C aro, C=N), 151.66 (C aro), 149.05 (C aro), 148.99 (C aro), 135.65 (C aro), 132.71 (C aro), 123.56 (C aro), 115.97 (2×C aro), 115.78 (2×C aro), 67.30 (CH_2_), 59.87 (CH_2_). MS *m/z*: 282.07 (M-1), 283.93 (M+1).

10g (1-(2-(3-((2H-tetrazol-5-yl)methoxy)phenoxy)ethyl)-1H-indole): yellowish solid (50.24%), m.p. 150.0-154.5°C, R_f_ = 0.39 (developing agent: dichloromethane / methanol = 10:1); ^1^H-NMR (DMSO-d_6_, 400MHz): δ 7.562-7.527 (2H, m, Ar-H); 7.423-7.415 (1H, d, *J* = 3.2 Hz, Ar-H); 7.200-7.141 (2H, m, Ar-H); 7.037-7.017 (1H, t, *J* = 4 Hz, Ar-H); 6.629-6.592 (2H, m, Ar-H); 6.586-6.527 (1H, m, Ar-H); 6.446 (1H, s, Ar-H); 5.422 (2H, s, CH_2_); 4.577-4.551 (2H, t, *J* = 5.2 Hz, CH_2_); 4.299-4.273 (2H, t, *J* = 5.2 Hz, CH_2_). ^13^C-NMR (DMSO-d_6_, 101MHz): δ 159.27 (2×C aro), 158.53 (C=N), 135.84 (C aro), 130.15 (C aro), 129.02 (C aro), 128.08 (=C-N), 121.00 (C aro), 120.32 (C aro), 118.98 (C aro), 109.91 (C aro), 107.83 (C aro), 107.38 (C aro), 101.52 (C aro), 100.72 (=CH), 66.97 (CH_2_), 59.30 (CH_2_), 44.94 (CH_2_). MS *m/z*: 334.13(M-1), 335.07(M), 336.13(M+1).

10h (9-(2-(3-((2H-tetrazol-5-yl)methoxy)phenoxy)ethyl)-9H-carbazole): white solid (10.70%), m.p. 199.8-202.1°C, R_f_ = 0.42 (developing agent: dichloromethane/methanol = 10:1); ^1^H-NMR (DMSO-d_6_, 400MHz): δ 8.155-8.136 (2H, d, *J* = 7.6 Hz, Ar-H); 7.693-7.672 (2H, d, *J* = 8.4 Hz, Ar-H); 7.484-7.443 (2H, m, Ar-H); 7.228-7.190 (2H, m, Ar-H); 7.161-7.120 (1H, t, *J* = 8.2 Hz, Ar-H); 6.602-6.576 (1H, m, Ar-H); 6.523-6.511 (1H, t, *J* = 2.4 Hz, Ar-H); 6.472-6.446 (1H, dd, *J* = 8.4 Hz, Ar-H); 5.391 (2H, s, CH_2_); 4.812-4.786 (2H, t, *J* = 5.2 Hz, CH_2_); 4.370-4.344 (2H, t, *J* = 5.2 Hz, CH_2_). ^13^C-NMR (DMSO-d_6_, 101MHz): δ 159.28 (C aro, C=N), 158.48 (C aro), 140.20 (2×C aro), 130.11 (C aro), 125.62 (2×C aro), 122.11 (2×C aro), 120.12 (2×C aro), 118.88 (2×C aro), 109.57 (2×C aro), 107.85 (C aro), 107.23 (C aro), 101.41 (C aro), 66.45 (CH_2_), 59.30 (CH_2_), 42.00 (CH_2_). MS *m/z*: 384.13 (M-1), 385.20 (M), 386.07 (M+1).

10i (1-(3-(3-((2H-tetrazol-5-yl)methoxy)phenoxy)propyl)-1H-indole): yellowish solid (42.17%), m.p. 112.5-116.1°C, R_f_ = 0.28 (developing agent: dichloromethane / methanol = 30:1); ^1^H-NMR (DMSO-d_6_, 400MHz): δ 7.546-7.527 (1H, d, *J* = 7.6 Hz, Ar-H); 7.479-7.458 (1H, d, *J* = 8.4 Hz, Ar-H); 7.352-7.344 (1H, d, *J* = 3.2 Hz, =CH); 7.226-7.183 (1H, t, *J* = 8.6 Hz, Ar-H); 7.118-7.080 (1H, t, *J* = 7.6 Hz, Ar-H); 7.099-7.081 (1H, d, *J* = 7.2 Hz, Ar-H); 7.019-7.000 (1H, d, *J* = 7.6 Hz, Ar-H); 6.652-6.561 (2H, m, Ar-H); 6.431-6.424 (1H, d, *J* = 2.8 Hz, =CH); 5.457 (2H, s, CH_2_); 4.364-4.330 (2H, t, *J* = 6.8 Hz, CH_2_); 3.894-3.864 (2H, t, *J* = 6.0 Hz, CH_2_); 2.225-2.160 (2H, m, CH_2_). ^13^C-NMR (DMSO-d_6_, 101MHz): δ 159.61 (C aro, C=N), 158.54 (C aro), 135.61 (C aro), 130.13 (C aro), 128.59 (C aro), 128.06 (=CH), 121.00 (C aro), 120.40 (C aro), 118.88 (C aro), 109.62 (C aro), 107.75 (C aro), 107.13 (C aro),101.68 (C aro), 100.63 (=CH), 64.62 (CH_2_), 59.28 (CH_2_), 42.16 (CH_2_), 30.65 (CH_2_). MS m/z: 348.13 (M-1), 350.13 (M+1), 372.13 (M+Na), 697.00 (2M-1), 720.67 (2M+Na).

10j (9-(3-(3-((2H-tetrazol-5-yl)methoxy)phenoxy)propyl)-9H-carbazole): white solid (34.79%), m.p. 157.0-160.3°C, R_f_ = 0.30 (developing agent: dichloromethane/methanol = 30:1); ^1^H-NMR (DMSO-d_6_, 400MHz): δ 7.600-7.580 (2H, d, *J* = 8.0 Hz, Ar-H); 7.421-7.380 (2H, m, Ar-H); 7.216-7.164 (3H, m, Ar-H); 6.999-6.981 (2H, d, *J* = 7.2 Hz, Ar-H); 6.650-6.628 (2H, m, Ar-H); 6.582-6.560 (1H, m, Ar-H); 5.454 (2H, s, CH_2_); 4.364-4.330 (2H, t, *J* = 6.8 Hz, CH_2_); 3.894-3.864 (2H, t, *J* = 6.0 Hz, CH_2_); 2.241-2.208 (2H, m, CH_2_). ^13^C-NMR (DMSO-d_6_, 101MHz): δ 159.55 (C aro, C=N), 158.53 (C aro), 139.92 (2×C aro), 130.10 (C aro), 125.67 (2×C aro), 122.06 (2×C aro), 120.24 (2×C aro), 118.74 (2×C aro), 109.07 (2×C aro), 107.72 (C aro), 107.13 (C aro), 101.73 (C aro), 64.63 (2×CH_2_), 59.28 (CH_2_), 30.65 (CH_2_). MS *m/z*: 398.13 (M-1), 797.00 (2M-1).

10k (5-((3-(3-Phenylpropoxy)phenoxy)methyl)-2H-tetrazole): white solid (44.36%), m.p. 121.1-124.9°C, R_f_ = 0.33 (developing agent: dichloromethane / methanol = 30:1); ^1^H-NMR (DMSO-d_6_, 400MHz): δ 7.306-7.270 (2H, m, Ar-H); 7.240-7.166 (4H, m, Ar-H); 6.636-6.593 (2H, m, Ar-H); 6.591-6.567 (1H, m, Ar-H); 5.459 (2H, s, CH_2_); 3.964-3.932 (2H, t, *J* = 6.4 Hz, CH_2_); 2.752-2.713 (2H, t, *J* = 7.8 Hz, CH_2_); 2.086-1.986 (2H, m, CH_2_). ^13^C-NMR (DMSO-d_6_, 101MHz): 159.82 (C aro, C=N), 158.57 (C aro), 141.32 (C aro), 130.11 (2×C aro), 128.31 (2×C aro), 125.81 (2×C aro), 107.77 (C aro), 107.03 (C aro), 101.66 (C aro), 66.73 (CH_2_), 59.29 (CH_2_), 31.43 (CH_2_), 30.65 (CH_2_). MS *m/z*: 309.07 (M-1), 310.20 (M), 311.07 (M+1).

10l (3-((3-((2H-tetrazol-5-yl)methoxy)phenoxy)methyl)pyridine): white solid (94.82%), m.p. 172.4-175.1°C, R_f_ = 0.36 (developing agent: dichloromethane/methanol = 30:1); ^1^H-NMR (DMSO-d_6_, 400MHz): δ 8.671 (1H, s, Ar-H); 8.558-8.548(1H, d, *J* = 4.0Hz, Ar-H); 7.878-7.859 (1H, d, *J* = 7.6Hz, Ar-H); 7.449-7.417 (1H, m, Ar-H); 7.261-7.220 (1H, t, *J* = 8.2 Hz, Ar-H); 6.751-6.657 (3H, m, Ar-H); 5.460 (2H, s, CH_2_); 5.145 (2H, s, CH_2_). ^13^C-NMR (DMSO-d_6_, 101MHz): δ 159.30 (2×C aro), 158.60 (C=N), 149.14 (C aro), 149.04 (C aro), 135.70 (C aro), 132.46 (C aro), 130.20 (C aro),123.59 (C aro), 108.05 (C aro), 107.45 (C aro), 102.06 (C aro), 66.97 (CH_2_), 59.34 (CH_2_). MS m/z: 241.13 (M+1), 263.07 (M+Na), 480.60 (M+1), 502.73 (2M+Na). MS *m/z*: 282.03(M-1), 283.03(M), 284.22(M+1), 305.98(M+Na).

### *In vitro* PPARα/γ/δ transactivation assays

PPARα agonist GW7647 and PPARδ agonist GW501516 were obtained from Sigma-Aldrich while PPARγ agonist rosiglitazone was from Zhejiang Hisun Pharmaceutical Company Limited. These reported agonists were regarded as positive controls (pc). The solvent DMSO with the same volume was added to the cell incubation solution and acted as the negative control (nc). The synthetic compounds and the positive controls were dissolved in DMSO. 293E cell and transfection reagents used in the biological assays were purchased from American type culture collection (ATCC) and Invitrogen Corporation, respectively.

Initially, synthetic molecules and the positive controls (10^−5^ M) were incubated with 293E cells which transfected with PPAR plasmid system (pcDNA3.1-hPPARLBD-Gal4DB and Peak12-6×Gal4UAS-luci) for 24 h. Then the values of normalized luciferase activities in cells incubated with synthetic molecules and controls were detected. With the activation folds of positive controls defined as the max, the relative activity (%) expressed as (mean±SEM) could be calculated as fomular (1):

Relative activity(%) = (Foldsample-Foldnc)(Foldpc-Foldnc)×100% (1)

Where sample means the synthetic compounds, pc and nc refer to the positive control and the negative control, respectively.

Based on the relative activity (% activity), six compounds (6e, 6g, 6h, 6l, 10h and 10l) were further screened with specific PPAR activity.

Furthermore, compounds 6h and GW7647 in multiple concentration gradients were incubated with 293E cells transfected with PPARα plasmid system (pcDNA3.1-hPPARαLBD-Gal4DB and Peak12-6×Gal4UAS-luci) for 24 h. Compounds 6g, 10h and rosiglitazone in multiple concentration gradients were incubated with 293E cells transfected with PPARγ plasmid system (pcDNA3.1-hPPARγLBD-Gal4DB and Peak12-6×Gal4UAS-luci) for 24 h. Compounds 6e, 6l, 10l and GW501516 in multiple concentration gradients were incubated with 293E cells transfected with PPARδ plasmid system (pcDNA3.1-hPPARδLBD-Gal4DB and Peak12-6×Gal4UAS-luci) for 24 h. The solvent DMSO with the same volume was added to the cell incubation solution and acted as the negative control. Then the values of normalized luciferase activities in cells incubated with these six molecules and controls in various concentrations could be detected. The relative activity (%) expressed as (mean±SEM) could be calculated as fomular (2):

Relative activity(%) = (Foldsample-Foldnc)(Foldmax-Foldnc)×100% (2)

Where sample means the synthetic compounds, max means the maximum activation fold of the synthetic compounds (Max %), nc refers to the negative control.

Through the concentration-activity curves by GraphPad Prism 6 tool, the median effective concentration (EC_50_) and concentrations at maximum efficiency percentage (C_max_) of molecules in activating PPARs would be deduced.

### Molecular docking

The crystal structures of PPARs (1I7G.pdb for PPARα [22], 2PRG.pdb [23] for PPARγ and 3GZ9.pdb for PPARδ [24], respectively) were retrieved from RCSB PROTEIN DATA BANK (RCSB PDB,http://www.rcsb.org/).

The reference structures of proteins were subjected to Protein Preparation Wizard (Prep Wiz) [25–27] panel implemented in Maestro v10.2 (Schrödinger, LLC, New York, 2015) for optimization in several steps. Preliminarily, the preparation was preprocessed to assign bond orders to all bonds, add hygrogens to all atoms, creat disulfide bonds within 3.2 Å, cap termini to fill up ACE (N-acetyl) and NMA (N-methyl amide) and delete all unrelated waters [28]. Hydrogen bonding network and the orientation of Asn, Gln and His residues were optimized with H-bond assignment section [29]. A restraint minimization of the protein structure was performed with the root mean square deviation (RMSD) regulated to 0.5 and the force field set to Optimize Potentials for Liquid Simulations 2005 (OPLS_2005) [30, 31].

Ligand preparation was calculated with the LigPrep panel (Schrödinger, LLC, New York, 2015) to produce the corresponding low-energy 3D structures [32–34], which generated possible ionization states (at target PH: 7.0+/−2.0) [31], stereoisomers, tautomers and ring conformations [28].

With the preparation of protein and molecules completed, SP (standard precision) option in Ligand Docking protocol was utilized to dock ligands into the enclosing box [35, 36], which was generated with the original ligand centered. Finally, the docking quality would be evaluated by the glide scores, the binding interactions and the matching degree to the active cavity [37].

### Molecular dynamics simulations

For detailed dynamics behaviour of small molecules, 20 ns molecular dynamics (MD) of best docked complexes were simulated with Desmond v4.3 suite (D.E. Shaw Research, New York, NY, 2015), running CentOS7.1 Linux operating system. The operation was mainly conducted on well-prepared structures in several phases [38]. Initially, a model system for generating a solvated system was built with the solvent model set to predefined SPC [33], box shape to orthorhombic and box size calculation to Buffer method through the System Builder panel [39, 40]. The protein was reoriented to minimize the box volume. As the prepared structure was charged, it was necessary to be neutralized with counter ions. Additionally, ionic strength was enhanced via adding default salt of 0.15 M sodium chloride (NaCl) [41]. 20 ns MD task that simulated Newtonian dynamics to the built model system was performed with the model system loaded from workspace. For modeling convenience, the recording interval of trajectory was set to 5.0 ps [42] and the recording interval of the checkpoint file improved to 2000 ps. The checkfile was used to restart the simulation if interrupted and should be saved infrequently. Furthermore, the stage to relax model system before simulation was critical in case that the model system built by the System Builder was not optimal [33].

Before the simulation officially started, several stages [43, 44] were performed to relax the model system. After the MD simulation successfully completed, Simulation Interaction Diagram and Simulation Event Analysis panels were used to run the results analysis and investigate the binding stability.

## CONCLUSION

Motivated by the concept of multi-targets agents to avoid of the severe side effects, 27 molecules with the main structural skeleton of 3- or 4-alkoxy substituted phenoxyl were designed and synthesized successfully based on the combination principle and the bioisosterism principle. Preliminary *in vitro* PPARα/γ/δ transactivation assays yielded six compounds with potential PPARs activation. With the aid of molecular docking and molecular dynamics simulation of 20 ns, the interaction modes and binding stability of the representatives with PPARs were validated visually. The potential structures could be valuable references with further optimization for better PPARs activities.

## SUPPLEMENTARY MATERIALS FIGURES AND TABLE





## Abbreviations

PPAR: peroxisome proliferators-activated receptors
T2DM: type 2 diabetes mellitus
MD: molecular dynamic
EC_50_: median effective concentration
C_max_: concentration at maximum efficiency percentage
LBD: ligand binding domain
PL contacts: protein-ligand contacts
m.p.: melting points
TLC: thin-layer chromatography
pc: positive control
nc: negative control
ATCC: American type culture collection
PDB: PROTEIN DATA BANK
Prep Wiz: Protein Preparation Wizard
ACE: N-acetyl
NMA: N-methyl amide
RMSD: root mean square deviation
OPLS_2005: Optimize Potentials for Liquid Simulations 2005
SP: standard precision
NVT: constant Number of particles, Volume and Temperature
NPT: constant Number of particles, Pressure and Temperature.
