# Supplementary material for: Design, sythesis and evaluation of a series of 3- or 4-alkoxy substituted phenoxy derivatives as PPARs agonists
Source: Oncotarget. 2017 Feb 8;8(13):20766–83. doi: 10.18632/oncotarget.15198 (PMC5400543; doi:10.18632/oncotarget.15198)
Supplement: Supplementary file 1 [file oncotarget-08-20766-s001.pdf]

Design, sythesis and evaluation of a series of 3- or 4-alkoxy substituted phenoxy derivatives as PPARs agonists

Supplementary Materials

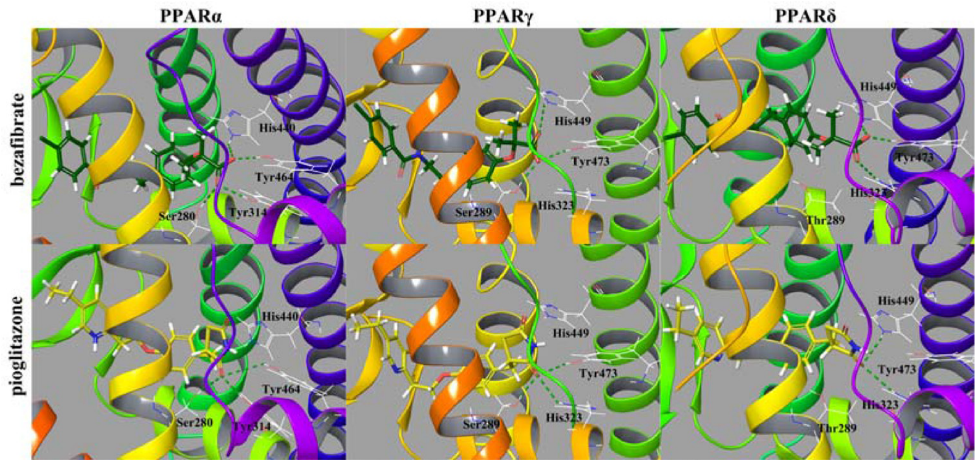

Supplementary Figure 1: The interaction behaviors between reference drugs (bezafibrate and pioglitazone) and PPARs active sites.

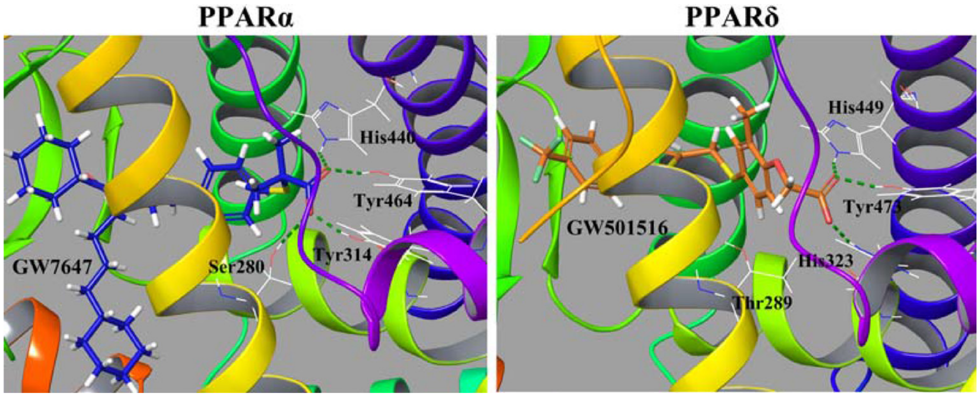

Supplementary Figure 2: The binding modes of GW7647 (PPARα agonist) and GW501516 (PPARδ agonist) with PPARα and PPARδ active sites, respectively.

Supplementary Table 1: Structures and *in vitro* preliminary screening of 3- or 4-alkoxy substituted phenoxy derivatives towards PPARs activation. see Supplementary_Table_1
