# Supplementary material for: Design, sythesis and evaluation of a series of 3- or 4-alkoxy substituted phenoxy derivatives as PPARs agonists
Source: Oncotarget. 2017 Feb 8;8(13):20766–83. doi: 10.18632/oncotarget.15198 (PMC5400543; doi:10.18632/oncotarget.15198)
Supplement: Supplementary file 2 [file oncotarget-08-20766-s002.docx]

**Supplementary Table 1** Structures and *in vitro* preliminary screening of 3- or 4-alkoxy substituted phenoxy derivatives towards PPARs activation.

| **Cpd#** | **Structure** | **PPARα** | | **PPARγ** | | **PPARδ** | |
| --- | --- | --- | --- | --- | --- | --- | --- |
|  |  | **Activation Fold^a^** | **Relative activity (%)^b^** | **Activation Fold** | **Relative activity (%)** | **Activation Fold** | **Relative activity (%)** |
| **6a** |  | 1.00±0.07 | 0.0±1.3 | 1.21±0.09 | 0.3±0.1 | 0.97±0.09 | (-0.3)±0.9 |
| **6b** |  | 0.80±0.00 | (-3.4)±0.0 | 1.19±0.00 | 0.3±0.0 | 1.23±0.07 | 2.4±0.7 |
| **6c** |  | 1.78±0.10 | 13.5±1.7 | 1.40±0.18 | 0.6±0.3 | 0.94±0.02 | (-0.6)±0.2 |
| **6d** |  | 1.96±0.17 | 16.6±3.0 | 1.57±0.00 | 0.8±0.0 | 1.13±0.03 | 1.4±0.3 |
| **6e** |  | 1.38±0.06 | 6.5±1.0 | 1.26±0.01 | 0.4±0.0 | 7.09±0.97 | 63.8±10.2 |
| **6f** |  | 1.58±0.09 | 10.0±1.6 | 1.22±0.01 | 0.3±0.0 | 1.27±0.09 | 2.8±1.0 |
| **6g** |  | 1.40±0.27 | 6.9±4.7 | 18.55±0.06 | 24.4±0.1 | 1.28±0.11 | 2.9±1.1 |
| **6h** |  | 3.81±0.83 | 48.5±14.4 | 1.57±0.25 | 0.8±0.3 | 0.78±0.15 | (-2.3)±1.5 |
| **6i** |  | 1.32±0.22 | 5.5±3.8 | 2.87±0.33 | 2.6±0.5 | 0.88±0.13 | (-1.3)±1.3 |
| **6j** |  | 0.97±0.15 | (-0.5)±2.6 | 1.17±0.00 | 0.2±0.0 | 0.86±0.03 | (-1.5)±0.3 |
| **6k** |  | 1.50±0.08 | 8.7±1.4 | 1.62±0.11 | 0.9±0.1 | 0.99±0.22 | (-0.1)±2.3 |
| **6l** |  | 1.39±0.21 | 6.7±3.5 | 1.34±0.01 | 0.5±0.0 | 6.60±0.90 | 58.6±9.4 |
| **6m** |  | 2.01±0.22 | 17.5±3.8 | 2.26±0.18 | 1.7±0.2 | 1.73±0.03 | 7.7±0.3 |
| **6n** |  | 1.16±0.02 | 2.8±0.4 | 1.31±0.12 | 0.4±0.2 | 1.24±0.06 | 2.5±0.6 |
| **6o** |  | 1.01±0.11 | 0.1±2.0 | 3.01±0.16 | 2.8±0.2 | 1.00±0.24 | 0.0±2.5 |
| **10a** |  | 0.76±0.04 | (-4.1)±0.7 | 1.30±0.06 | 0.4±0.1 | 1.28±0.06 | 2.9±0.6 |
| **10b** |  | 0.55±0.03 | (-7.8)±0.6 | 1.26±0.22 | 0.4±0.3 | 1.39±0.21 | 4.1±2.2 |
| **10c** |  | 0.67±0.02 | (-5.7)±0.3 | 0.96±0.01 | (-0.1)±0.0 | 1.01±0.08 | 0.1±0.9 |
| **10d** |  | 0.50±0.04 | (-8.6)±0.6 | 0.96±0.02 | (-0.1)±0.0 | 1.15±0.22 | 1.6±2.3 |
| **10e** |  | 1.91±0.13 | 15.7±2.2 | 1.93±0.23 | 1.3±0.3 | 1.26±0.15 | 2.7±1.6 |
| **10f** |  | 0.80±0.02 | (-3.5)±0.3 | 1.24±0.07 | 0.3±0.1 | 1.02±0.09 | 0.2±0.9 |
| **10g** |  | 0.90±0.05 | (-1.8)±0.8 | 2.83±0.49 | 2.5±0.0 | 0.83±0.04 | (-1.8)±0.5 |
| **10h** |  | 0.81±0.02 | (-3.3)±0.3 | 26.81±1.56 | 35.8±2.2 | 1.16±0.05 | 1.6±0.5 |
| **10i** |  | 0.89±0.19 | (-1.9)±3.3 | 1.19±0.09 | 0.3±0.1 | 0.77±0.01 | (-2.4)±0.1 |
| **10j** |  | 0.65±0.00 | (-6.0)±0.1 | 0.69±0.01 | (-0.4)±0.0 | 0.88±0.09 | (-1.2)±1.0 |
| **10k** |  | 0.74±0.09 | (-4.6)±1.5 | 1.46±0.20 | 0.6±0.3 | 1.24±0.17 | 2.5±1.8 |
| **10l** |  | 1.15±0.06 | 2.5±1.0 | 1.07±0.03 | 0.1±0.0 | 9.56±2.50 | 89.7±26.2 |
| **GW7647** |  | 6.80±1.93 | 100.0±33.3 | - | - | - | - |
| **rosiglitazone** |  | - | - | 73.01±3.79 | 100.0±5.3 | - | - |
| **GW501516** |  | - | - | - | - | 10.55±0.99 | 100.0±10.4 |
| **Negative Control** | DMSO | 1.00±0.18 | 0.0±3.2 | 1.00±0.06 | 0.0±0.1 | 1.00±0.03 | 0.0±0.3 |

a: activation fold: C_samples_ / C_control_, represented as (mean±SEM); b: the relative activity (% activity) towards PPARs compared to the positive controls.
